# Which adiposity index is best? Comparison of five indicators and their ability to identify type 2 diabetes risk in a population study

**DOI:** 10.1016/j.diabres.2025.112268

**Published:** 2025-05-20

**Authors:** Cunrong Huang, Andre Lopes, Annie Britton

**Affiliations:** a Research Department of Epidemiology and Public Health, University College London, United Kingdom; b Cancer Research UK & Cancer Trials Centre, University College London, United Kingdom

**Keywords:** Obesity, Adiposity index, Body mass index, Waist-to-height ratio, Type 2 diabetes

## Abstract

**Aims:**

We compared ability of five adiposity indicators [body mass index (BMI), waist circumference (WC), waist-to-height ratio (WHtR), waist-by-height^0.5^ (WHT.5R), and a body shape index (ABSI)] to identify current diabetes and their prospective associations with diabetes.

**Methods:**

Baseline data were from 7,979 participants of UK Whitehall II study, of whom 7,488 diabetes-free participants were followed-up (median = 16.0 years) for incident diabetes (n = 940). According to five indices’ cut-points, participants were separately classified into low-value groups and high-value groups. We cross-sectionally investigated ability of the indicators to identify existing diabetes by receiver operating characteristic curve analysis, and explored prospective associations between elevated indices and diabetes using Cox regression analysis.

**Results:**

Waist-based indicators were superior to BMI in identifying diabetes. High WHtR (≥0.5) demonstrated the highest multivariable-adjusted HR [2.64 (95 % CI 2.29, 3.03)]. Across all indicators, associations between elevated indicators and diabetes were stronger in younger participants. In combined analyses, “low BMI but high WHtR” had higher risk for diabetes [2.20 (95 % CI 1.65, 2.95)] than “high BMI but low WHtR” [1.34 (95 % CI 1.05, 1.70)].

**Conclusion:**

Waist-based indicators are more strongly associated with diabetes than BMI. WHtR, an easy-to-calculate, waist-based index with a sex- and race-independent cut-point, may be useful for diabetes prevention.

## 1. Introduction

Obesity, traditionally defined as an excess of body fat leading to impaired health, is a global public health epidemic [1]. For example, in the UK, it is predicted that around 35 % of all adults will be obese by 2025 [2]. Some observational studies suggest that obesity, associated with adipose dysfunction and insulin resistance, increases type 2 diabetes risk [3,4].

Body mass index (BMI), as an index of body weight in kilograms divided by height in square meter (kg/m^2^), is the most commonly used method to assess obesity in clinical practice with relevant guidelines (i.e., 25 to 30 kg/m^2^ of BMI is overweight; ≥30 kg/m^2^ is obese) [5,6], and has been widely accepted as a quick and simple tool to classify patients into different risk categories [7]. However, BMI does not distinguish fat from muscle or between different body fat distributions [8]. Additionally, it has been observed that individuals with a more central (or abdominal) fat distribution are at greater health risk than those with peripheral fat [9], thus, when we look at obesity, we cannot ignore the corresponding risk associated with elevated waist circumference (WC) while considering excessive body weight (i.e., BMI). Furthermore, several studies indicated that some indices related to abdominal obesity, such as WC or waist-to-height ratio (WHtR), may more accurately predict diabetes risk than BMI [10,11]. Compared with WC, WHtR is an index of abdominal obesity corrected for height, and its recognized cut-off value is 0.5 (<0.5 is considered normal), regardless of sex or ethnicity [12]. Besides BMI and WC, a number of novel adiposity indicators for defining obesity continue to emerge. For example, waist-by-height^0.5^ (WHT.5R) is a new indicator developed in 2016 [13], which apportions less importance to height than WHtR. WHT.5R has been shown to better predict cardiometabolic risks, including high lipids and blood glucose, than BMI [13]. A body shape index (ABSI), proposed in 2012, was specifically developed as a transformation of WC, with minimal correlation to height, weight, or BMI [14]. Complementary to BMI, the primary goal of this index is to capture the excess risk attributed to elevated WC. It was initially used to predict premature mortality risk [15]. However, these two newly-developed indices do not yet have accepted cut-points like BMI and WHtR, and at present, there are limited studies that simultaneously compare traditional indicators and these new ones with respect to their ability to discriminate current or future diabetes risk.

Furthermore, fat distribution is influenced by factors such as sex and age, which means that these relevant characteristics should be taken into account when applying anthropometry to assess an individual’s health risk. Some studies suggested the existence of sex differences and age differences in the strength of association between adiposity indices and diabetes [16–18]. For example, a cross-sectional study of 35,256 Chinese adults aged 20–74 years showed that the association between elevated adiposity indicators and diabetes was stronger in their younger participants [18]. This suggests that stratification by sex and age is necessary when investigating the association between obesity and diabetes. However, the potential differences in sex and age remain inconclusive because there is limited biological evidence that can explain them and results of relevant studies were not entirely consistent [19]. In addition, few studies examined multiple (>3) obesity indicators along with investigating multiple important relevant stratification factors, and it remains unclear which adiposity index is more appropriate for assessing diabetes risk in specific demographic groups (such as women or men). In order to better understand and utilize different adiposity indicators, we conducted corresponding stratification analyses.

In this large, population-based study, we aimed to compare the ability of five adiposity indices (BMI, WC, WHtR, WHT.5R, and ABSI) to cross-sectionally identify individuals with diabetes, and to compare their prospective associations with incident diabetes, in order to assess future diabetes risk among those without diabetes at baseline.

## 2. Methods

### 2.1. Data collection

This study used data from the UK Whitehall II (WHII) study. Briefly, the WHII is an ongoing cohort study of 10,308 British civil servants aged 35–55 years, of whom 3,413 (33.1 %) were females, at initial enrollment (Phase 1), recruited from 20 London-based offices during 1985–1988 [20,21]. Phase 1 of WHII included a clinical examination and a self-administered questionnaire to collect data covering demographics, health status and lifestyle factors. Subsequent phases of information collection have alternated between questionnaire alone and questionnaire accompanied by a clinical examination [22,23]. The University College London ethics committee approved the study. Use of human material conformed to the Declaration of Helsinki.

### 2.2. Subjects

**Fig. 1.**
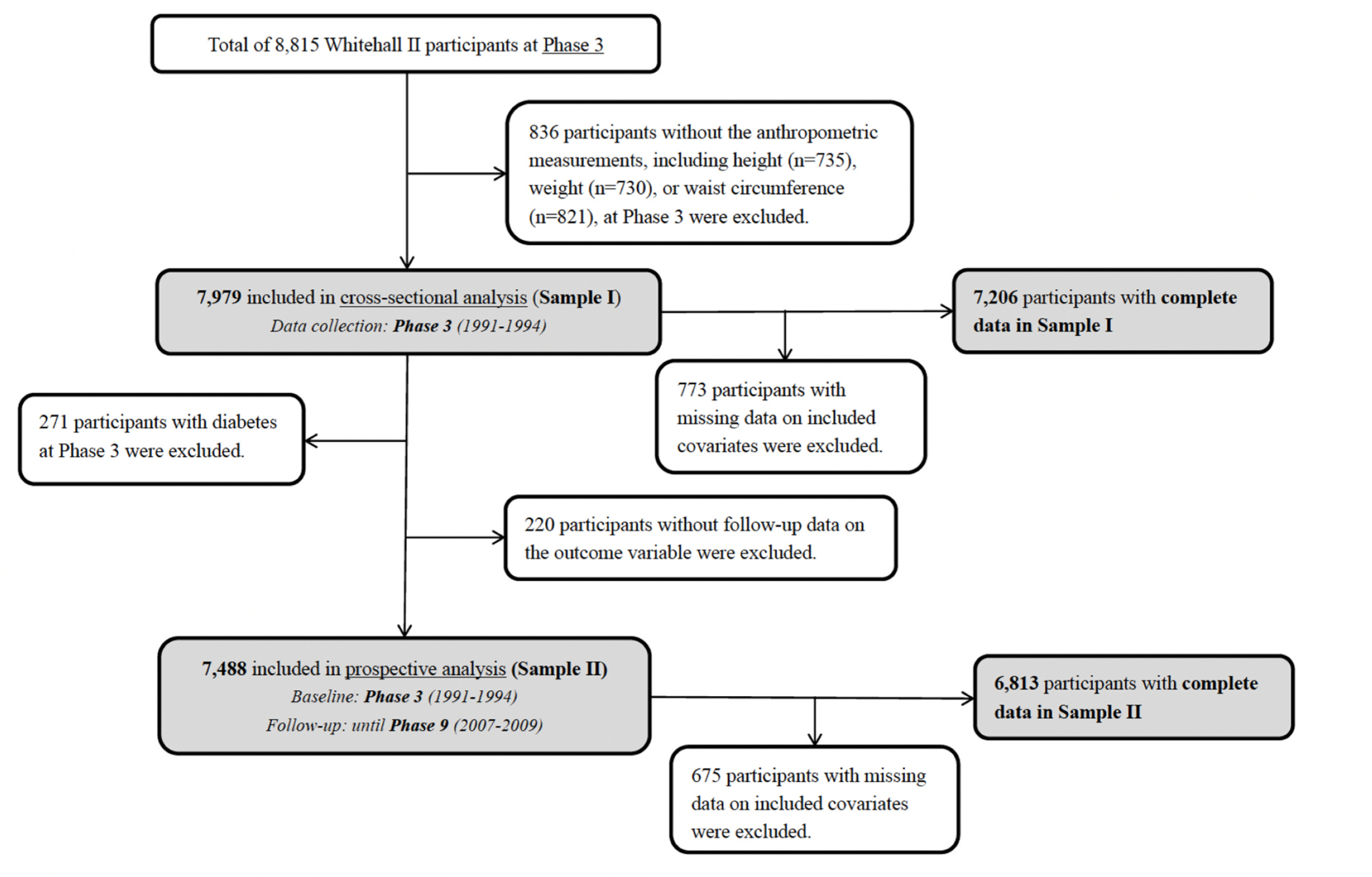
Flow chart of the included participants.

For the purposes of the present study, Phase 3 of the Whitehall II study (1991–1994) was considered the baseline, as it was the first phase to collect anthropometric measurements. Participants without data on height, weight, or waist circumference at Phase 3 were excluded. The remaining participants constituted the cross-sectional study sample (Sample I), which was used to compare the ability of different adiposity indices to identify individuals with diabetes at baseline. For the prospective analyses, participants with diabetes at Phase 3 were further excluded to form Sample II, which was used to examine the prospective association between adiposity indices and incident diabetes during follow-up until Phase 9 (2007–2009), the most recent diabetes screening phase. A flow chart of sample construction is shown in Fig. 1.

### 2.3. Assessments

The exposures are five adiposity indicators, including body mass index (BMI) [weight (kg)/height^2^ (m^2^)], waist circumference (WC) [cm], waist-to-height ratio (WHtR) [WC (cm)/height (cm)], waist-by-height^0.5^ (WHT.5R) [WC (cm)/Height^0.5^ (cm^0.5^)], and a body shape index (ABSI) [WC (m)/(BMI^2/3^ (kg/m^2^)^2/3^ × height^1/2^ (m^1/2^))]. Of these, weight was measured with all items of clothing removed except underwear, using an electronic Soehnle scale with a digital readout. Height was measured with a stadiometer with the individual standing erect with the head in the Frankfort plane, and WC was measured using a fiberglass tape measure at 600 g tension as the smallest circumference at or below the costal margin [24].

To enable quantitative comparison of the exposures, we used standardized values of the exposures in cross-sectional analyses. For the longitudinal part of the analyses, we dichotomized the adiposity indicators according to their cut-points. For indicators with an accepted cut-point, we chose that recognized value, and for indicators without an accepted cut-off value, we selected the 75th population-wide percentile as its cut-point [5,12,25,26]. That is, Sample II were separately divided into low-value group (reference group) [BMI: <25 kg/m^2^; WC: <90 cm for men and < 80 cm for women; WHtR: <0.5; WHT.5R < 75th population-wide centile (6.911 cm^0.5^); ABSI < 75th population-wide percentile (0.078 m^7/6^/kg^2/3^), respectively] and high-value group (≥corresponding cut-off value), i.e., different adiposity indicators were used as different criteria to classify individuals into low or high level of obesity-related risk. To ensure comparability of different exposures and minimize the loss of numerical information when converting to categorical variables, in the longitudinal part, we additionally analyzed the associations of the standardized exposure variables (i.e., continuous) with diabetes risk.

Subgroup analyses were based on sex (male or female), age (<50 or ≥ 50 years, median age), ethnicity (white or non-white), and baseline cardiovascular disease (CVD) diagnosis (yes or no) for investigating differences in the strength of association between elevated adiposity indices and diabetes among different stratifications. For further examining health effect of different categories of obesity, such as excessive weight alone, abdominal obesity alone or both, we consider individuals’ weight and WC at the same time. According to the different levels of BMI and WHtR combination, the Sample II was subdivided into 4 groups: (1) low BMI (<25 kg/m^2^) and low WHtR (< 0.5) (reference group), (2) high BMI (≥25 kg/m^2^) and low WHtR (< 0.5), (3) low BMI (< 25 kg/m^2^) and high WHtR (≥0.5), (4) high BMI (≥25 kg/m^2^) and high WHtR (≥0.5).

Potential confounding factors were age (continuous), sex (female or male), ethnicity (white or non-white), smoking (never smoker, ex-smoker, or current smoker), drinking (current not drinking, current moderate, current heavy), socioeconomic position (high, intermediate or low) [22], baseline CVD diagnosis (yes or no), CVD medication (yes or no), family history of diabetes (yes or no), physical activity (active, moderately active, or inactive) [27], dietary behavior (frequency of fruit and vegetables consumed; <daily or ≥ daily) [22], and menopause status (female subjects only: yes or no).

The outcome, type 2 diabetes, was defined as having a fasting plasma glucose ≥ 7.0 mmol/L, a 2-hour postload glucose ≥ 11.1 mmol/L at clinical examination, a physician diagnosis of type 2 diabetes, use of diabetes medication, or a hospital record of diabetes between 1991 and 2009 [28,29]. For each participant, follow-up time began on the date that anthropometric measurements were taken in Phase 3 and ended on the occurrence of diabetes, death, emigration, or the end date of last survey, whichever occurred first.

### 2.4. Statistical analyses

Continuous variables were presented as means ± standard deviations (SDs) or median (25th–75th percentiles), and categorical variables as frequencies with proportions. Differences between groups were assessed using two-sided t-tests or Wilcoxon rank-sum tests for continuous variables, and chi-squared tests for categorical variables. Correlations between the five adiposity indicators were estimated using Pearson’s correlation coefficient (r).

In cross-sectional analyses, receiver operating characteristic (ROC) curves were constructed for each of the five indices, and the area under the ROC curve (AUC) was calculated to identify the index with the highest AUC as the strongest discriminator for diabetes. For each index, AUCs were calculated using two models: one without covariates and one with the inclusion of age, sex, ethnicity, smoking, drinking, socioeconomic position, physical activity, dietary behavior, family history of diabetes, baseline CVD diagnosis, and CVD medication. In both models, apart from the adiposity index being evaluated, all other variables included in the model were kept the same to ensure a fair comparison across indices. These covariates were treated as potential confounders, rather than predictive variables, to specifically evaluate the independent discriminatory contribution of each adiposity measure.

In cohort analyses, Cox regression analysis was used to estimate hazard ratios (HRs) and 95 % confidence interval (CI) for time to diabetes in relation to the elevated adiposity indices. For each adiposity index separately, three Cox models were estimated. Model_1_ did not include any covariate. Adjusted covariates for the Model_2_ included age, sex, and ethnicity. For the Model_3_, besides the covariates of the Model_2_, we additionally included smoking, drinking, socioeconomic position, baseline CVD diagnosis, CVD medication, family history of diabetes, physical activity, and dietary behavior. The differences in cumulative event (diabetes) rates were evaluated based on different combinations of BMI and WHtR, using log-rank test, and its results were presented as Kaplan-Meier (K-M) curves. Additionally, we did two-by-two comparisons between the four groups and used the Benjamini-Hochberg method to adjust the significance level. We also used Cox regression analysis to estimate HRs and 95 % CI for diabetes in relation to different levels of BMI and WHtR combination. Proportional hazards assumption was tested based on Schoenfeld residuals. P values of the global test indicated that the assumption was not entirely met. However, according to the K-M curves for the exposures (Supplementary Fig. S1) and the graphs of the scaled Schoenfeld residuals against the transformed time (Supplementary Fig. S2), the violation of proportionality was not extreme, which means a single HR for the exposure can still be a reasonable summary of the data [30].

To address potential bias due to missingness in included covariates (Supplementary Table S1), we conducted multivariate imputation (MI) by chained equations, using 30 imputed data sets. All reported P values were 2-sided. All analyses were performed in R (version 4.4.1).

## 3. Results

### 3.1. Characteristics of participants

The details of participants’ baseline characteristics are shown in Supplementary Table S2. A total of 7,979 individuals were available for the cross-sectional analysis (Sample I). In Sample I, the mean age was 50.06 ± 6.03 years and 2,468 (30.9 %) were female. In the cohort part, there were 7,488 persons (Sample II). Further details (baseline characteristics of groups based on different adiposity indicators and participants with complete records) are provided in Supplementary Table S3 and Supplementary Table S4. When the study population was stratified by sex, significant differences were observed in adiposity indices and several demographic and lifestyle characteristics. Men had a larger mean WC and a higher mean WHtR, whereas women had a higher mean BMI and a higher mean age (Supplementary Table S5a and S5b).

### 3.2. Correlation analysis

BMI, WC, WHtR, and WHT.5R were strongly correlated with each other and with weight (all r > 0.7). WHtR is the only one of our indicators that is essentially independent of height (r close to 0). ABSI was strongly correlated with WC (r > 0.7) but weakly correlated with BMI (r < 0.3), whereas its correlation with WHtR (r was around 0.6) and WHT.5R (r was around 0.7) was moderately strong (Supplementary Table S6).

### 3.3. ROC curve analyses

Of 7,979 participants included in the baseline visit, 271 (3.4 %) had baseline diabetes. Results of ROC curves analyses without inclusion of covariates demonstrated WHtR had a higher AUC (0.638) than other indicators (Table 1). After including covariates, there was a general improvement in the discriminatory ability of our adiposity indicators in identifying diabetes (all AUCs > 0.7). Although the differences in AUCs between the 5 indicators were small, WHtR was the indicator with the largest AUC in imputed data (0.728). For complete data (Supplementary Table S7), ABSI had the highest AUC (0.720). The AUC for BMI was consistently lower than the AUCs for the other 4 measures. When the study population was stratified by sex, waist-based measures consistently showed higher AUCs than BMI in both males and females. WHtR had the highest AUC among males, while WHtR and ABSI shared the highest AUC among females. Overall, AUCs were higher in males than in females across all adiposity indices (Supplementary Table S8).

### 3.4. Cox regression analyses

Of 7,488 participants included in the cohort analyses, after a median follow-up of 16.0 years (interquartile range: 14.2–16.5 yrs), 940 individuals developed diabetes, and of those 940, complete covariate data were available for 847. The estimated HRs associated with elevated adiposity indices (high-value group) are shown in Table 2. In all the models, higher levels of adiposity indicators were associated with a greater risk of developing diabetes. In the maximally-adjusted models, WHtR had a relatively higher HR (complete data: 2.67, 95 % CI: 2.31–3.10; imputed data: 2.64, 95 % CI: 2.29–3.03) for incident diabetes than the other indicators. The HRs for the waist-based measures (WC, WHtR, and WHT.5R) were all higher than the HR of BMI. Moreover, when analyses were performed using standardized values of the exposures (continuous), our results still showed that waist-based measures had larger HRs for diabetes than BMI (Supplementary Table S9).

**Fig. 2.**
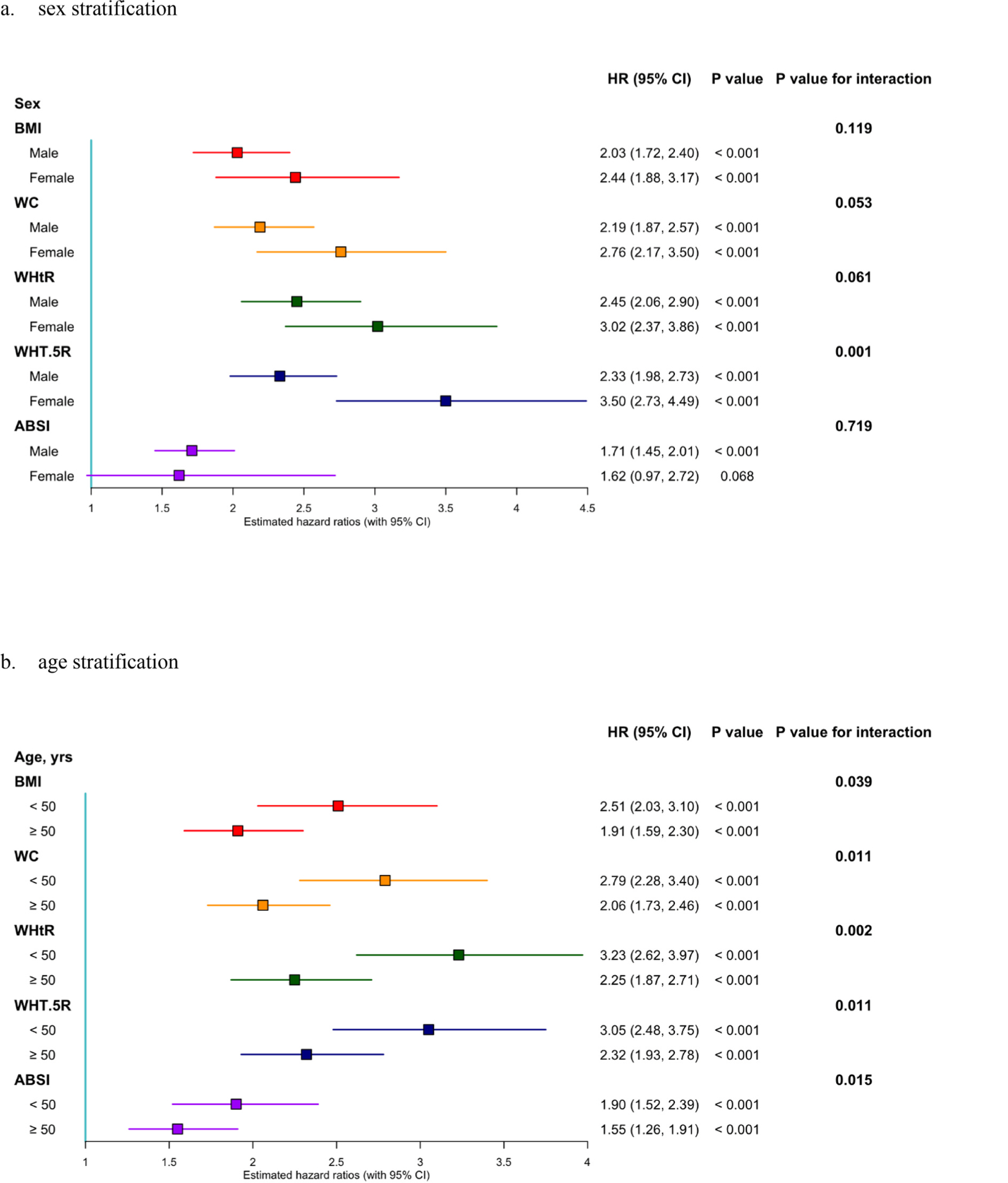

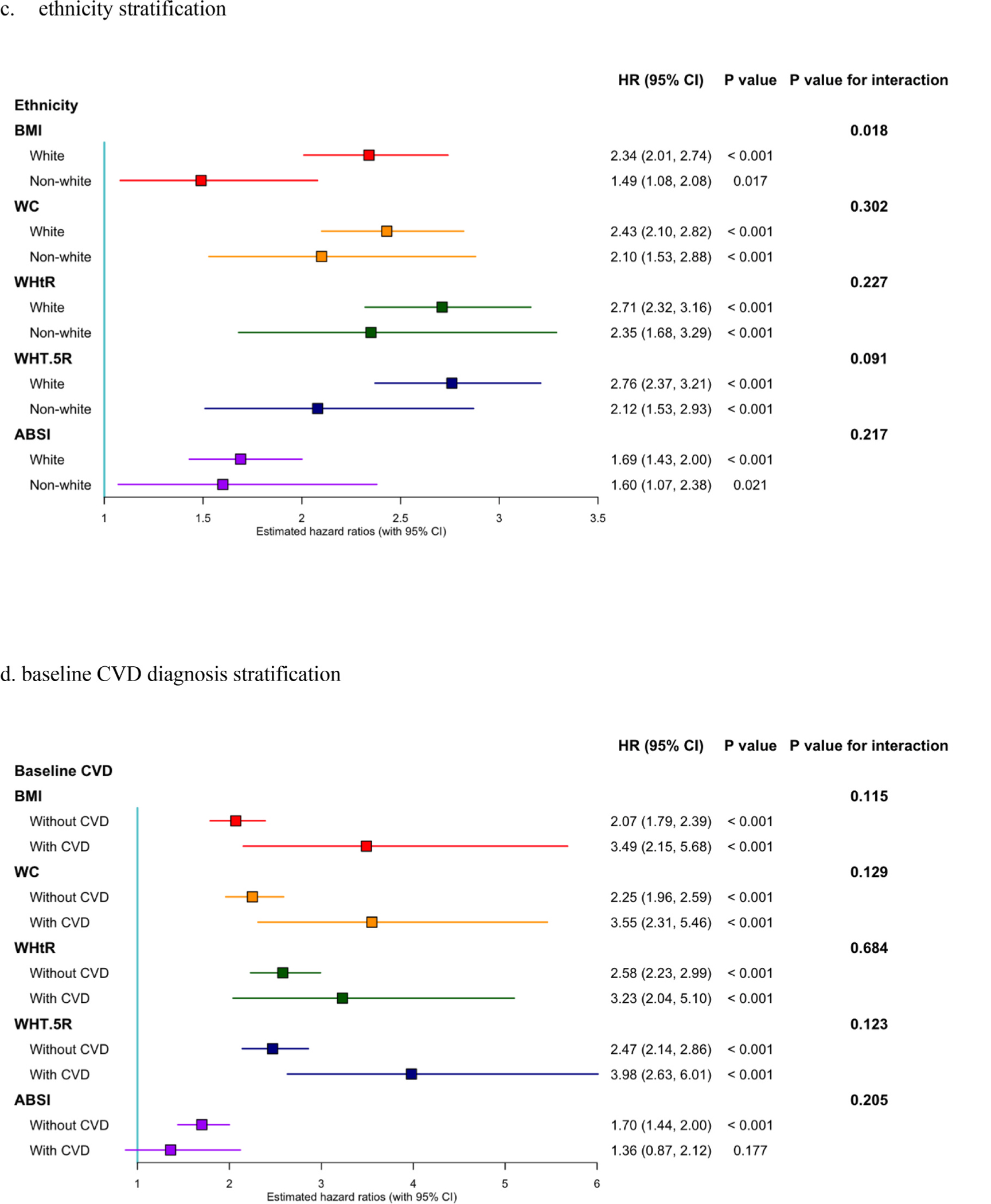
Subgroup analyses for the associations between the five indices (high-value group) and incident diabetes in Sample II. BMI, body mass index; WC, waist circumference; WHtR, waist-to-height ratio; WHT.5R, waist-by-height^0.5^; ABSI, a body shape index; HR, hazard ratio; CI, confidence interval; CVD, cardiovascular disease.

Fig. 2 illustrates the results of subgroup analyses to identify potential modifiers of the associations between the five indices and incident diabetes in Sample II. We found strong statistical evidence for effect modification only for age 50 (P value for interaction < 0.05), whereby the association between each adiposity indicator and diabetes was stronger in people aged under 50 at baseline, compared with those aged 50 and older. Additionally, in both age groups, elevated WHtR and WHT.5R had the highest HRs for diabetes, suggesting the strong association of the two indices with diabetes. Regarding the other potential modifiers we considered, generally, among females, the white, and persons with baseline CVD, elevated adiposity indicators except ABSI appear to confer a higher risk increase of developing diabetes than males, the non-white, and individuals without baseline CVD, but the observed differences are small in most cases, and the statistical evidence for most interactions is weak. More information is shown in Supplementary Table S10 and Supplementary Table S11.

### 3.5. Combining BMI and WHtR as metrics for diabetes risk

Supplementary Fig. S3a and S3b show Kaplan-Meier plots for time to diabetes event, stratified by different levels of BMI and WHtR combination. Comparisons between the groups showed strong evidence of differences in incidence of diabetes between the groups (all P < 0.001) except for “low BMI and high WHtR” and “high BMI and high WHtR” (Supplementary Table S12), which was not statistically significant according to the Benjamini-Hochberg procedure (P > 0.05). Hence, it was inferred that the cumulative diabetes rate in the “low BMI and high WHtR” group was significantly higher than that in the “high BMI and low WHtR” group. Meanwhile, in Cox regression analysis (Table 3), the “low BMI and high WHtR” group also showed a higher diabetes risk than the “high BMI and low WHtR” group in both the complete (HR_2_: 2.34 vs 1.48) and imputed data (HR_2_: 2.20 vs 1.34).

## 4. Discussion

This study explored the ability of different adiposity indicators to cross-sectionally identify existing diabetes, as well as their prospective association with incident diabetes. The results showed that waist-based measures were superior to BMI in identifying current diabetes. Although all elevated adiposity indices were associated with a higher incident diabetes risk, the waist-based measures had stronger associations than BMI. Persons with low BMI but high WHtR had a higher risk of developing diabetes than individuals with high BMI but low WHtR. This study provided information on the capacity of waist-based indicators to identify diabetes risk, providing the evidence of the rationale for routine monitoring of waist-based indicators in general practice, in addition to BMI.

First, in the cross-sectional analyses, we investigated the ability of different adiposity indicators to identify existing diabetes, and found that waist-based measures performed better than BMI, even though the differences are small, which is consistent with some studies [11,31]. For example, a *meta*-analysis including 39 relevant studies showed WC and WHtR were better at discriminating diabetes than BMI [11]. The explanation may be in part due to abdominal fat distribution is a more important risk factor for diabetes than general obesity [32]. Compared with BMI, waist-based measures can better reflect the accumulation of abdominal fat or ectopic fat. Additionally, although the AUCs given by WC and WHT.5R were almost as high as that of WHtR, the AUC of WHtR was slightly higher with or without controlling for covariates. It may be because WHtR is the only one of the three waist-based measures that is essentially uncorrelated with height according to our correlation analyses (height and WHtR: r was close to 0), which means it may reflect better on individual abdominal fat and reduce the influence of height on the results to some extent. As for ABSI, after including covariates, its AUC increased considerably, being only lower than WHtR in the imputed data and slightly higher than WHtR in the complete data. It suggested that for our study population, ABSI is no less capable of discriminating current diabetes than other waist-based measures, and better than BMI. However, ABSI has disadvantages for clinical applications: it is complex to compute and due to its small absolute value, individual differences in values are usually not distinguishable until 3 or 4 decimal places. Additionally, we found that, in both sexes, waist-based adiposity indices generally had higher AUCs than BMI, which is consistent with our findings in the overall population. While AUCs were higher in men than in women across all adiposity indices, this difference may be partly explained by sex-related differences in fat distribution and metabolic risk [33]. At a given adiposity level, men tend to have a higher proportion of visceral fat than women, which is strongly associated with diabetes risk [34]. Furthermore, type 2 diabetes is often diagnosed at a younger age and lower BMI in men than in women [35], which may suggest that equivalent levels of adiposity indices correspond to a higher immediate diabetes risk in men. These differences might contribute to the higher discriminative performance (AUC) of adiposity indices observed in men in this study.

For prospective association between the elevated adiposity indicators and diabetes, we found that elevated waist-based measures had higher HRs for incident diabetes than BMI, which is consistent with some studies [16,36]. For instance, a *meta*-analysis including 15 eligible studies with 120,012 persons indicated that WHtR and WC had a greater association with diabetes than BMI [36]. As mentioned before, abdominal obesity, which is highly associated with visceral fat [37], is a more important risk factor compared with general obesity, thus, measures of abdominal obesity, such as WC, WHtR, and WHT.5R, were more strongly associated with diabetes risk than BMI. Meanwhile, waist-based measures reflect not only visceral fat but also subcutaneous abdominal fat, which is strongly associated with insulin resistance and diabetes [38]. Additionally, our study showed high-value group by WHtR cut-point had the highest HR for incident diabetes, with a 42 % higher risk of developing diabetes than high-value group by BMI cut-off, indicating that “0.5 of WHtR” appears to be a better public health tool for classifying individuals into obesity-related diabetes risk categories than the often-used BMI cut-off of 25 kg/m^2^. Although ABSI was developed as a transformation of WC, and increased ABSI was strongly associated with incident diabetes in our study, it produced a relatively weaker association than the three waist-based measures of this study, which is broadly in agreement with some studies [16,39]. The nature of the variables used in the ABSI calculation and the relationships between the variables may be a potential explanation for the results. ABSI was proposed to associate body shape with mortality, statistically independently of BMI [14]. However, our outcome is diabetes, not death. Additionally, a study showed that ABSI was weakly correlated with metabolic syndrome and cardiometabolic risk which are highly associated with diabetes [40]. Furthermore, although our findings showed that increased WC seems to be more strongly associated with the diabetes than increased BMI, it does not imply that the effect of BMI gain on diabetes can be disregarded. Hence, the weak correlation of ABSI with BMI may also contribute to the current results.

In stratification analyses of prospective associations, although we have not found many statistically significant interactions between elevated adiposity indices and sex, age, ethnicity, or baseline CVD diagnosis for diabetes, overall, our measures (except ABSI) had relatively higher absolute HRs in women and relatively younger participants. These findings may be related to the following points. (1) The observation that elevated adiposity indicators were more strongly associated with diabetes risk in women is consistent with some studies [16,41]. We speculate that this may be because women tend to have a higher body fat percentage than men and fat distribution varies by sex [33]. An increase in WC appears to have a greater effect on diabetes risk than an equivalent gain in weight. When women are overweight or obese, they preferentially deposit fat subcutaneously [42], and their excess subcutaneous fat may contribute more to increased WC than in men [41], potentially elevating their diabetes risk. Additionally, the majority of the women in our study were either postmenopausal (50.9 % in our data) or currently perimenopausal. During this period, abdominal fat accumulation, especially visceral fat, becomes more pronounced in females [43]. Consequently, the association between visceral adipose tissue and diabetes risk in women is likely to become stronger, indicating their higher future diabetes risk compared with males. However, sex difference is still controversial. For example, a relevant study suggested the association between increased adiposity indices and diabetes risk was generally stronger in men [17]. Currently, there is no clear biological mechanism to explain the potential sex difference. (2) In our study, we found a weaker association between obesity and diabetes in older people (50 + years) compared with younger people, suggesting that measuring adiposity indicators may be particularly useful in the relatively younger individuals in our data. A similar finding was reported in a related study [18]. However, the reason for this finding remains unclear. We hypothesized that it may be because age is a strong influencing factor for diabetes, which may lead to a high risk of developing the disease. Consequently, the association between adiposity indicators and diabetes may be correspondingly reduced in the older age group. Further research with a broader age range is warranted. (3) Notably, our study had relatively low precision in estimating the HRs for non-white individuals and those with baseline CVD due to the small sample size (<10 %) in these subgroups.

Although central-obesity indices are thought to be more strongly associated with diabetes than BMI, we cannot completely ignore the influence of weight gain, and among our three waist-based measures, WHtR, a height-adjusted measure of WC with an accepted single cut-point, is more stable as it is less influenced by race and sex. Therefore, we further studied the effects of different levels of BMI and WHtR combinations on diabetes development, and found that “low BMI but high WHtR” group (4.7 % of sample II; “inaccurately” classified as low obesity-related risk by BMI criteria alone) had a higher risk of developing diabetes than the “high BMI but low WHtR” group (classified as high obesity-related risk by BMI criteria alone), which suggests excessive WC may have a greater effect on diabetes development than abnormal BMI. This result also indicates that when assessing diabetes risk based on obesity (or overweight status), we should not rely solely on BMI being within the normal range, as individuals with normal BMI but elevated WHtR are also at high diabetes risk. A cross-sectional study of 46,979 participants reported similar results, providing support to our findings [44]. Therefore, more people may benefit from including “maintaining normal waist-based indicators in addition to BMI” in their health management.

Even though our baseline (1991–1994) and follow-up (2007–2009) data are historical, it is well documented that the prevalence of both obesity and type 2 diabetes has continued to rise substantially from the early 1990s to the present [2,45]. These long-term trends suggest that a middle-aged individual today may face a higher absolute risk of developing diabetes than a counterpart during our study period. However, the relative associations between adiposity indices and diabetes risk are thought to be broadly consistent over time, which may be explained by stable underlying biological mechanisms [46]. Therefore, compared with BMI, the observed greater discriminative ability and stronger association of waist-based measures (such as WHtR) in relation to diabetes risk are likely to remain applicable in present-day clinical and public health settings.

There are some limitations in this study. First, our study used only a single measurement of the adiposity indicators, assuming it represents adulthood; however, these indices may change during the follow-up period. Hence, our future research will further explore this topic using repeated measurements of adiposity indicators. Second, when doing stratification analyses, the sample size for some subgroups, such as non-whites and individuals with baseline CVD, was small. This implies low statistical power to detect differences between subgroups and reduced precision in the estimates. Moreover, the predominance of White participants (over 90 %) may limit the generalizability of the findings to other populations, particularly those with markedly different ethnic compositions. Third, participants with a single fasting glucose measurement ≥ 7.0 mmol/L or 2-hour postload glucose ≥ 11.1 mmol/L, without confirmation by a second test, were classified as having diabetes, which may have led to some degree of misclassification. In addition, hemoglobin A1c (HbA1c) was not included in the diagnostic criteria, as it was not collected until Phase 7 in the WHII. Forth, data on fat content and distribution were not available for Phase 3 of WHII. Thus, we were unable to directly investigate the association between the adiposity indices and body fat or visceral fat. Fifth, since there are no recognized cut-offs for WHT.R and ABSI, we chose the 75th percentile as their cut-points [47]; however, the values (i.e., 75th percentile) may vary when the study population changes. However, we also analyzed the association between standardized values of the five indices and diabetes (avoiding some drawbacks of a dichotomous approach), reaffirming that waist-based measures are more strongly associated with diabetes risk than BMI.

## 5. Conclusion

This study adds evidence that waist-based measures may be more strongly associated with diabetes risk than BMI. As an easy-to-calculate, WC-derived index with a sex- and race-independent cut-point, WHtR monitoring in general practice, alongside BMI, may play a role in diabetes prevention.

## Supplementary Material

1

## Figures and Tables

**Table 1:** Area under the receiver operating characteristic curve for five adiposity indicators in relation to diabetes in the Sample I.

Exposures	AUC_1_ (95 % CI) Sample I: no covariates	AUC_2_ (95 % CI) Sample I: with covariates
**BMI**	0.588 (0.552, 0.624)	0.722 (0.718, 0.726)
**WC**	0.604 (0.568, 0.640)	0.724 (0.720, 0.728)
**WHtR**	0.638 (0.603, 0.674)	0.728 (0.724, 0.732)
**WHT.5R**	0.624 (0.588, 0.660)	0.726 (0.722, 0.730)
**ABSI**	0.604 (0.569, 0.640)	0.727 (0.723, 0.731)

AUC_1_, among Sample I (sample size = 7,979, number of events = 271), without covariates;

AUC_2_, among Sample I (sample size =7,979, number of events =271), including covariates for age, sex, ethnicity, smoking, drinking, socioeconomic position, physical activity, dietary behavior, family history of diabetes, baseline CVD diagnosis, CVD medication, and imputation for the missing covariates;

AUC, area under the curve; CVD, cardiovascular disease; CI, confidence interval; BMI, body mass index; WC, waist circumference; WHtR, waist-to-height ratio; WHT.5R, waist-by-height^0.5^; ABSI, a body shape index.

**Table 2:** Estimated hazard ratios for incident diabetes in relation to the five adiposity indices (reference: low-value group).

	N ^a^ (%)	Diabetes, ^a^ N (%)	Crude HR_0_, ^a^ (95 % CI)	Adjusted HR_1_, ^a^ (95 % CI)	Adjusted HR_2_, ^a^ (95 % CI)	N ^b^ (%)	Diabetes, ^b^ N (%)	Adjusted HR_2_ with MI, ^b^ (95 % CI)
**BMI**								
Low	3629 (53.3)	277 (7.6)	1	1	1	3949 (52.7)	310 (7.9)	1
High	3184 (46.7)	570 (17.9)	2.54 (2.20, 2.93)	2.41 (2.09, 2.79)	2.25 (1.94, 2.60)	3539 (47.3)	630 (17.8)	2.17 (1.89, 2.49)
**WC**								
Low	4497 (66.0)	377 (8.4)	1	1	1	4908 (65.5)	415 (8.5)	1
High	2316 (34.0)	470 (20.3)	2.69 (2.35, 3.08)	2.56 (2.23, 2.93)	2.37 (2.06, 2.72)	2580 (34.5)	525(20.3)	2.36 (2.07, 2.70)
**WHtR**								
Low	4171 (61.2)	310 (7.4)	1	1	1	4557 (60.9)	344 (7.5)	1
High	2642 (38.8)	537 (20.3)	3.04 (2.64, 3.50)	2.88 (2.49, 3.33)	2.67 (2.31, 3.10)	2931 (39.1)	596 (20.3)	2.64 (2.29, 3.03)
**WHT.5R**								
Low	5167 (75.8)	471 (9.1)	1	1	1	5666 (75.7)	518 (9.1)	1
High	1646 (24.2)	376 (22.8)	2.78 (2.43, 3.19)	2.77 (2.40, 3.18)	2.54 (2.21, 2.93)	1822 (24.3)	422 (23.2)	2.63 (2.29. 3.01)
**ABSI**								
Low	5140 (75.4)	546 (10.6)	1	1	1	5655 (75.5)	612 (10.8)	1
High	1673 (24.6)	301 (18.0)	1.80 (1.56, 2.07)	1.87 (1.60, 2.20)	1.70 (1.45, 2.00)	1833 (24.5)	328 (17.9)	1.67 (1.43, 1.94)

BMI: low: <25 kg/m^2^, high: ≥25 kg/m^2^; WC: low: <90 cm for men and < 80 cm for women; high: ≥90 cm for men and ≥ 80 cm for women; WHtR: low: <0.5; high: ≥0.5; WHT.5R: low: <75th population-wide centile (6.911 cm^0.5^), high: ≥75th population-wide centile (6.911 cm^0.5^); ABSI: low: <75th population-wide percentile (0.078 m^7/6^/kg^2/3^), high: ≥75th population-wide percentile (0.078 m^7/6^/kg^2/3^);

Crude HR_0_, the Cox regression model did not include any covariate (Model 1);

Adjusted HR_1_, the Cox regression model was adjusted for age, sex, and ethnicity (Model 2);

Adjusted HR_2_, the Cox regression model was adjusted for age, sex, ethnicity, smoking, drinking, socioeconomic position, physical activity, dietary behavior, family history of diabetes, baseline CVD diagnosis, and CVD medication (Model 3);

Adjusted HR_2_ with MI, the imputed Cox regression model was adjusted for age, sex, ethnicity, smoking, drinking, socioeconomic position, physical activity, dietary behavior, family history of diabetes, baseline CVD diagnosis, and CVD medication;

MI, multivariate imputation; CVD, cardiovascular disease; HR, hazard ratio; CI, confidence interval; BMI, body mass index; WC, waist circumference; WHtR, waist-to-height ratio; WHT.5R, waist-by-height^0.5^; ABSI, a body shape index.

a Using complete data (n = 6,813) of Sample II, number of events for complete data = 847;

b Using Sample II, sample size = 7,488, number of events = 940, imputation for the missing covariates.

**Table 3:** Crude and adjusted hazard ratios for diabetes in relation to combination of BMI and WHtR (reference: low BMI and low WHtR).

Groups	N ^a^ (%)	Diabetes, ^a^ N (%)	Crude HR_0_, ^a^ (95 % CI)	Adjusted HR_1_, ^a^ (95 % CI)	Adjusted HR_2_, ^a^ (95 % CI)	N^b^ (%)	Diabetes, ^b^ N (%)	Adjusted HR2 with MI, ^b^ (95 % CI)
**low BMI and low WHtR**	3299 (48.3)	218 (6.6)	1	1	1	3597 (48.0)	248 (6.9)	1
**high BMI and low WHtR**	872 (13.0)	92 (10.6)	1.67 (1.31, 2.13)	1.57 (1.23, 2.00)	1.48 (1.16, 1.90)	960 (12.8)	96 (10.0)	1.34 (1.05, 1.70)
**low BMI and high WHtR**	330 (4.7)	59 (17.9)	3.12 (2.34, 4.15)	2.56 (1.90, 3.45)	2.34 (1.73, 3.15)	352 (4.7)	62 (17.6)	2.20 (1.65, 2.95)
**high BMI and high WHtR**	2312 (34.0)	478 (20.7)	3.50 (2.98, 4.10)	3.30 (2.80, 3.88)	3.03 (2.57, 3.57)	2579 (34.4)	534 (20.7)	2.90 (2.49, 3.39)

Crude HR_0_, the Cox regression model did not include any covariate (Model 1);

Adjusted HR_1_, the Cox regression model was adjusted for age, sex, and ethnicity (Model 2);

Adjusted HR_2_, the Cox regression model was adjusted for age, sex, ethnicity, smoking, drinking, socioeconomic position, physical activity, dietary behavior, family history of diabetes, baseline CVD diagnosis, and CVD medication (Model 3);

Adjusted HR_2_ with MI, the imputed Cox regression model was adjusted for age, sex, ethnicity, smoking, drinking, socioeconomic position, physical activity, dietary behavior, family history of diabetes, baseline CVD diagnosis, and CVD medication;

MI, multivariate imputation; CVD, cardiovascular disease; HR, hazard ratio; CI, confidence interval; BMI, body mass index; WHtR, waist-to-height ratio.

a Using complete data (n = 6,813) of Sample II, number of events for complete data = 847;

b For Sample II, sample size = 7,488, number of events = 940, imputation for the missing covariates.

## Appendix A. Supplementary data

Supplementary data to this article can be found online at https://doi.org/10.1016/j.diabres.2025.112268.
