# Supplementary material for: Which adiposity index is best? Comparison of five indicators and their ability to identify type 2 diabetes risk in a population study
Source: Diabetes Res Clin Pract. Author manuscript; Available in PMC 2026 Jul 28. (PMC13408304; doi:10.1016/j.diabres.2025.112268)
Supplement: 1 [file NIHMS2176164-supplement-1.docx]

**Supplementary Table S1** Missingness in the included covariates for the participants

|  | **Sample I (7979)**  **Missing, N (%)** | **Sample II (7488)**  **Missing, N (%)** |
| --- | --- | --- |
| **Age** | 0 (0) | 0 (0) |
| **Sex** | 0 (0) | 0 (0) |
| **Ethnicity** | 28 (0.4) | 20 (0.3) |
| **Smoking** | 630 (7.9) | 543 (7.3) |
| **Drinking** | 174 (2.2) | 131 (1.7) |
| **Socioeconomic position** | 173 (2.2) | 129 (1.7) |
| **Physical activity** | 167 (2.1) | 124 (1.7) |
| **Dietary behavior** | 172 (2.2) | 129 (1.7) |
| **CVD diagnosis** | 0 (0) | 0 (0) |
| **CVD medication** | 0 (0) | 0 (0) |
| **Family history of diabetes** | 122 (1.5) | 116 (1.5) |
| **Menopause status (female)** | 60/2468 (2.4) | 42/2295 (1.8) |

CVD, cardiovascular disease

**Supplementary Table S2** Characteristics of the participants in Phase 3 (baseline time)

| Characteristic | Sample I, N (%)  N = 7979 (100) | **Sample II, N (%)**  **N = 7488 (100)** |
| --- | --- | --- |
| Baseline Diabetes, N (%) | 271/7979 (3.4) | 0/7488 (0) |
| Age, mean ± SD, yrs | 50.06 ± 6.03 | 49.95 ± 6.01 |
| < 50 | 4231/7979 (53.0) | 4035/7488 (53.9) |
| ≥ 50 | 3748/7979 (47.0) | 3453/7488 (46.1) |
| Sex, N (%) |  |  |
| Male | 5511/7979 (69.1) | 5193/7488 (69.4%) |
| Female | 2468/7979 (30.9) | 2295/7488 (30.6) |
| Ethnicity, N (%) |  |  |
| White | 7188/7951 (90.4) | 6827/7468 (91.4) |
| Non-white | 763/7951 (9.6) | 641/7468 (8.6) |
| Height, mean ± SD, cm | 171.86 ± 9.45 | 172.01 ± 9.4 |
| Weight, mean ± SD, kg | 74.88 ± 12.74 | 74.83 ± 12.67 |
| BMI, mean ± SD, kg/m^2^ | 25.32 ± 3.74 | 25.26 ± 3.68 |
| WC, mean ± SD, cm | 83.82 ± 11.49 | 83.65 ± 11.41 |
| WHtR, mean ± SD | 0.49 ± 0.06 | 0.49 ± 0.06 |
| WHT.5R, mean ± SD, cm^0.5^ | 6.39 ± 0.83 | 6.38 ± 0.82 |
| ABSI, mean ± SD, m^7/6^/kg^2/3^ | 0.07 ± 0.01 | 0.07 ± 0.01 |
| SBP, mean ± SD, mm Hg | 120.70 ± 13.63 | 120.47 ± 13.53 |
| DBP, mean ± SD, mm Hg | 79.75 ± 9.41 | 79.66 ± 9.38 |
| TC, mean ± SD, mmol/L | 6.49 ± 1.15 | 6.48 ± 1.16 |
| TG, mean ± SD, mmol/L | 1.49 ± 1.13 | 1.46 ± 1.10 |
| LDL-C, mean ± SD, mmol/L | 4.39 ± 1.03 | 4.39 ± 1.04 |
| HDL-C, mean ± SD, mmol/L | 1.43 ± 0.41 | 1.44 ± 0.41 |
| Fasting glucose, mean ± SD, mmol/L | 5.24 ± 0.69 | 5.20 ± 0.47 |
| CRP, ^a^ median (IQR), mg/L | 0.89 (0.45, 1.90) | 0.87 (0.44, 1.83) |
| IL-6, ^a^ median (IQR), pg/mL | 1.41 (1.03, 2.07) | 1.39 (1.02, 2.03) |
| Baseline CVD diagnosis, N (%) |  |  |
| Yes | 738/7979 (9.2) | 682/7488 (9.1) |
| No | 7241/7979 (90.8) | 6806/7488 (90.9) |
| **Family history of CVD**  **(angina, MI, stroke), N (%)** | 3671/7039 (52.2) | 3450/6603 (52.2) |
| **Family history of diabetes, N (%)** | 906/7857 (11.5) | 799/7372 (10.8) |
| **Family history of hypertension, N (%)** | 3077/6855 (44.9) | 2891/6427 (45.0) |
| **Smoking Status, N (%)** |  |  |
| Never smoker | 3452/7349 (47.0) | 3286/6945 (47.3) |
| Ex-smoker | 2839/7349 (38.6) | 2680/6945 (38.6) |
| Current smoker | 1058/7349 (14.4) | 979/6945 (14.1) |
| **Drinking, N (%)** |  |  |
| Current not drinking | 1513/7805 (19.4) | 1378/7357 (18.7) |
| Current moderate (1-14 unit/w) | 4399/7805 (56.4) | 4178/7357 (56.8) |
| Current heavy (>14 unit/w) | 1893/7805 (24.3) | 1801/7357 (24.5) |
| **Fruit/vegetable consumption, N (%)** |  |  |
| < Daily | 3040/7807 (38.9) | 2840/7359 (38.6) |
| ≥ Daily | 4767/7807 (61.1) | 4519/7359 (61.4) |
| **Physical activity** |  |  |
| Inactive | 1610/7812 (20.6) | 1467/7364 (19.9) |
| Moderate | 2780/7812 (35.6) | 2649/7364 (36.0) |
| Active | 3422/7812 (43.8) | 3248/7364 (44.1) |
| **Education level, N (%)** |  |  |
| Low | 300/5989 (5.0) | 284/5619 (5.1) |
| Middle | 3120/5989 (52.1) | 2909/5619 (51.8) |
| High | 2569/5989 (42.9) | 2426/5619 (43.2) |
| **Socioeconomic position, N (%)** |  |  |
| Low | 1297/7806 (16.6) | 1143/7359 (15.5) |
| Intermediate | 2973/7806 (38.1) | 2872/7359 (39.0) |
| High | 3536/7806 (45.3) | 3344/7359 (45.4) |

^a^ Not follow normal distribution, shown with median (25th–75th percentiles);

SD, standard deviation; IQR, interquartile range; BMI, body mass index; WC, waist circumference; WHtR, waist-to-height ratio; WHT.5R, waist-by-height^0.5^; ABSI, a body shape index; CVD, cardiovascular disease; MI, myocardial infarction; TC, total cholesterol; TG, triglycerides; LDL-C, low-density lipoprotein cholesterol; HDL-C, high-density lipoprotein cholesterol; SBP, systolic blood pressure; DBP, diastolic blood pressure; BP, blood pressure; CRP, C-Reactive Protein; IL-6, Interleukin 6;

Drinking: current moderate (1-14 unit/w), current heavy (>14 unit/w); Socioeconomic position: defined using either current or last recorded employment grade; Physical activity: active (>2.5 hrs/week of moderate physical activity or >1 hr/week of vigorous physical activity), inactive (<1 hr/week of moderate physical activity and <1 hr/week of vigorous physical activity), or moderately active (if not active or inactive).

**Supplementary Table S3a** Characteristics of the Sample II in Phase 3 *according to BMI criterion*

| **Characteristic** | **BMI < 25 kg/m^2^, N (%)**  **N = 3949 (52.7)** | **BMI ≥ 25 kg/m^2^, N (%)**  **N = 3539 (47.3)** | **P value ^a^** |
| --- | --- | --- | --- |
| **Age, mean ± SD, yrs** | 49.48 ± 6.00 | 50.47 ± 5.98 | < 0.001 |
| **Sex (Female), N (%)** | 1196/3949 (30.3) | 1099/3539 (31.1) | 0.487 |
| **Ethnicity (White), N (%)** | 3643/3936 (92.6) | 3184/3532 (90.1) | < 0.001 |
| **Height, mean ± SD, cm** | 172.53 ± 9.30 | 171.44 ± 9.49 | < 0.001 |
| **Weight, mean ± SD, kg** | 67.67 ± 9.09 | 82.81 ± 11.24 | < 0.001 |
| **SBP, mean ± SD, mm Hg** | 117.84 ± 13.33 | 123.40 ± 13.15 | < 0.001 |
| **DBP, mean ± SD, mm Hg** | 77.33 ± 9.01 | 82.25 ± 9.10 | < 0.001 |
| **TC, mean ± SD, mmol/L** | 6.32 ± 1.11 | 6.66 ± 1.18 | < 0.001 |
| **TG, mean ± SD, mmol/L** | 1.22 ± 0.91 | 1.73 ± 1.22 | < 0.001 |
| **LDL-C, mean ± SD, mmol/L** | 4.24 ± 1.01 | 4.55 ± 1.04 | < 0.001 |
| **HDL-C, mean ± SD, mmol/L** | 1.53 ± 0.42 | 1.34 ± 0.37 | < 0.001 |
| **Fasting glucose, mean ± SD, mmol/L** | 5.14 ± 0.46 | 5.26 ± 0.47 | < 0.001 |
| **CRP, ^b^ median (IQR), mg/L** | 0.63 (0.32, 1.25) | 1.22 (0.66, 2.53) | < 0.001 |
| **IL-6, ^b^ median (IQR), pg/mL** | 1.25 (0.93, 1.78) | 1.58 (1.15, 2.35) | < 0.001 |
| **Baseline CVD diagnosis, N (%)** | 304/3949 (7.7) | 378/3539 (10.7) | < 0.001 |
| **Family history of CVD**  **(angina, MI, stroke), N (%)** | 1782/3480 (51.2) | 1668/3123 (53.4) | 0.078 |
| **Family history of diabetes, N (%)** | 359/3902 (9.2) | 440/3470 (12.7) | < 0.001 |
| **Family history of hypertension, N (%)** | 1493/3387 (44.1) | 1398/3040 (46.0) | 0.131 |
| **Smoking Status, N (%)** |  |  | < 0.001 |
| Never smoker | 1835/3687 (49.8) | 1451/3258 (44.5) |  |
| Ex-smoker | 1333/3687 (36.2) | 1347/3258 (41.3) |  |
| Current smoker | 519/3687 (14.1) | 460/3258 (14.1) |  |
| **Drinking, N (%)** |  |  | < 0.001 |
| Current not drinking | 718/3902 (18.4) | 660/3455 (19.1) |  |
| Current moderate (1-14 unit/w) | 2292/3902 (58.7) | 1886/3455 (54.6) |  |
| Current heavy (>14 unit/w) | 892/3902 (22.9) | 909/3455 (26.3) |  |
| **Fruit/vegetable consumption, N (%)** |  |  | < 0.001 |
| < Daily | 3040/3900 (36.4) | 1420/3459 (41.1) |  |
| ≥ Daily | 4767/3900 (63.6) | 2039/3459 (58.9) |  |
| **Physical activity** |  |  | < 0.001 |
| Inactive | 1610/3903 (17.9) | 1467/3461 (22.2) |  |
| Moderate | 2780/3903 (36.6) | 2649/3461 (35.3) |  |
| Active | 3422/3903 (45.5) | 3248/3461 (42.6) |  |
| **Education level, N (%)** |  |  | < 0.001 |
| Low | 133/2932 (4.5) | 151/2687 (5.1) |  |
| Middle | 1409/2932 (48.1) | 1500/2687 (51.8) |  |
| High | 1390/2932 (47.4) | 1036/2687 (43.2) |  |
| **Socioeconomic position, N (%)** |  |  | < 0.001 |
| Low | 510/3902 (13.1) | 633/3457 (18.3) |  |
| Intermediate | 1646/3902 (42.2) | 1226/3457 (35.5) |  |
| High | 1746/3902 (44.7) | 1598/3457 (46.2) |  |

^a^ T-test or Wilcoxon rank-sum test for continuous variables and chi-square test for categorical variables;

^b^ Not follow normal distribution, shown with median (25th-75th percentiles);

BMI, body mass index; SD, standard deviation; IQR, interquartile range; CVD, cardiovascular disease; MI, myocardial infarction; TC, total cholesterol; TG, triglycerides; LDL-C, low-density lipoprotein cholesterol; HDL-C, high-density lipoprotein cholesterol; SBP, systolic blood pressure; DBP, diastolic blood pressure; BP, blood pressure; CRP, C-Reactive Protein; IL-6, Interleukin 6

**Supplementary Table S3b** Characteristics of the Sample II in Phase 3 *according to WC criterion*

| **Characteristic** | **WC < cut-point, N (%)**  **N = 4908 (65.5)** | **WC ≥ cut-point, N (%)**  **N = 2580 (34.5)** | **P value ^a^** |
| --- | --- | --- | --- |
| **Age, mean ± SD, yrs** | 49.47 ± 5.99 | 50.85 ± 5.94 | < 0.001 |
| **Sex (Female), N (%)** | 1603/4908 (32.7) | 692/2580 (26.8) | < 0.001 |
| **Ethnicity (White), N (%)** | 4504/4892 (92.1) | 2323/2576 (90.2) | 0.006 |
| **Height, mean ± SD, cm** | 171.38 ± 9.29 | 173.22 ± 9.51 | < 0.001 |
| **Weight, mean ± SD, kg** | 69.04 ± 9.25 | 85.84 ± 10.86 | < 0.001 |
| **SBP, mean ± SD, mm Hg** | 118.15 ± 13.15 | 124.89 ± 13.14 | < 0.001 |
| **DBP, mean ± SD, mm Hg** | 77.66 ± 8.96 | 83.46 ± 8.98 | < 0.001 |
| **TC, mean ± SD, mmol/L** | 6.36 ± 1.13 | 6.71 ± 1.17 | < 0.001 |
| **TG, mean ± SD, mmol/L** | 1.23 ± 0.87 | 1.90 ± 1.33 | < 0.001 |
| **LDL-C, mean ± SD, mmol/L** | 4.28 ± 1.02 | 4.58 ± 1.03 | < 0.001 |
| **HDL-C, mean ± SD, mmol/L** | 1.52 ± 0.42 | 1.29 ± 0.36 | < 0.001 |
| **Fasting glucose, mean ± SD, mmol/L** | 5.15 ± 0.46 | 5.28 ± 0.46 | < 0.001 |
| **CRP, ^b^ median (IQR), mg/L** | 0.67 (0.35, 1.32) | 1.40 (0.78, 2.87) | < 0.001 |
| **IL-6, ^b^ median (IQR), pg/mL** | 1.26 (0.94, 1.81) | 1.69 (1.22, 2.52) | < 0.001 |
| **Baseline CVD diagnosis, N (%)** | 370/4908 (7.5) | 312/2580 (12.1) | < 0.001 |
| **Family history of CVD**  **(angina, MI, stroke), N (%)** | 2213/4334 (51.2) | 1237/2269 (53.4) | 0.008 |
| **Family history of diabetes, N (%)** | 458/4847 (9.4) | 341/2525 (13.5) | < 0.001 |
| **Family history of hypertension, N (%)** | 1879/4246 (44.3) | 1012/2181 (46.4) | 0.107 |
| **Smoking Status, N (%)** |  |  | < 0.001 |
| Never smoker | 2276/4569 (49.8) | 1010/2376 (42.5) |  |
| Ex-smoker | 1657/4569 (36.3) | 1023/2376 (43.1) |  |
| Current smoker | 636/4569 (13.9) | 343/2376 (14.4) |  |
| **Drinking, N (%)** |  |  | < 0.001 |
| Current not drinking | 913/4847 (18.8) | 465/2510 (18.5) |  |
| Current moderate (1-14 unit/w) | 2865/4847 (59.1) | 1313/2510 (52.3) |  |
| Current heavy (>14 unit/w) | 1069/4847 (22.1) | 732/2510 (29.2) |  |
| **Fruit/vegetable consumption, N (%)** |  |  | < 0.001 |
| < Daily | 1771/4845 (36.6) | 1069/2514 (42.5) |  |
| ≥ Daily | 3074/4845 (63.4) | 1445/2514 (57.5) |  |
| **Physical activity** |  |  | < 0.001 |
| Inactive | 889/4848 (18.3) | 578/2516 (23.0) |  |
| Moderate | 1725/4848 (35.6) | 924/2516 (36.7) |  |
| Active | 2234/4848 (46.1) | 1014/2516 (40.3) |  |
| **Education level, N (%)** |  |  | < 0.001 |
| Low | 178/3684 (4.8) | 106/1935 (5.5) |  |
| Middle | 1829/3684 (49.6) | 1080/1935 (55.8) |  |
| High | 1677/3684 (45.5) | 749/1935 (38.7) |  |
| **Socioeconomic position, N (%)** |  |  | 0.005 |
| Low | 708/4847 (14.6) | 435/2512 (17.3) |  |
| Intermediate | 1934/4847 (39.9) | 938/2512 (37.3) |  |
| High | 2205/4847 (45.5) | 1139/2512 (45.3) |  |

^a^ T-test or Wilcoxon rank-sum test for continuous variables and chi-square test for categorical variables;

^b^ Not follow normal distribution, shown with median (25th-75th percentiles);

WC, waist circumference; WC cut-point: 90 cm for men and 80 cm for women; SD, standard deviation; IQR, interquartile range; CVD, cardiovascular disease; MI, myocardial infarction; TC, total cholesterol; TG, triglycerides; LDL-C, low-density lipoprotein cholesterol; HDL-C, high-density lipoprotein cholesterol; SBP, systolic blood pressure; DBP, diastolic blood pressure; BP, blood pressure; CRP, C-Reactive Protein; IL-6, Interleukin 6.

**Supplementary Table S3c** Characteristics of the Sample II in Phase 3 *according to WHtR criterion*

| **Characteristic** | **WHtR < 0.5, N (%)**  **N = 4557 (60.9)** | **WHtR ≥ 0.5, N (%)**  **N = 2931 (39.1)** | **P value ^a^** |
| --- | --- | --- | --- |
| **Age, mean ± SD, yrs** | 49.26 ± 5.95 | 51.01 ± 5.94 | < 0.001 |
| **Sex (Female), N (%)** | 1646/4557 (36.1) | 649/2931 (22.1) | < 0.001 |
| **Ethnicity (White), N (%)** | 4232/4543 (93.2) | 2595/2925 (88.7) | 0.006 |
| **Height, mean ± SD, cm** | 172.17 ± 9.61 | 171.77 ± 9.07 | 0.072 |
| **Weight, mean ± SD, kg** | 69.30 ± 9.88 | 83.43 ± 11.68 | < 0.001 |
| **SBP, mean ± SD, mm Hg** | 117.91 ± 13.09 | 124.45 ± 13.25 | < 0.001 |
| **DBP, mean ± SD, mm Hg** | 77.38 ± 8.89 | 83.19 ± 9.03 | < 0.001 |
| **TC, mean ± SD, mmol/L** | 6.31 ± 1.13 | 6.73 ± 1.15 | < 0.001 |
| **TG, mean ± SD, mmol/L** | 1.19 ± 0.86 | 1.88 ± 1.28 | < 0.001 |
| **LDL-C, mean ± SD, mmol/L** | 4.24 ± 1.02 | 4.62 ± 1.02 | < 0.001 |
| **HDL-C, mean ± SD, mmol/L** | 1.53 ± 0.42 | 1.28 ± 0.35 | < 0.001 |
| **Fasting glucose, mean ± SD, mmol/L** | 5.14 ± 0.46 | 5.29 ± 0.47 | < 0.001 |
| **CRP, ^b^ median (IQR), mg/L** | 0.64 (0.34, 1.27) | 1.36 (0.74, 2.79) | < 0.001 |
| **IL-6, ^b^ median (IQR), pg/mL** | 1.26 (0.93, 1.80) | 1.64 (1.18, 2.43) | < 0.001 |
| **Baseline CVD diagnosis, N (%)** | 332/4557 (7.3) | 350/2931 (11.9) | < 0.001 |
| **Family history of CVD**  **(angina, MI, stroke), N (%)** | 2045/4019 (50.9) | 1405/2584 (54.4) | 0.006 |
| **Family history of diabetes, N (%)** | 419/4495 (9.3) | 380/2877 (13.2) | < 0.001 |
| **Family history of hypertension, N (%)** | 1756/3936 (44.6) | 1135/2491 (45.6) | 0.471 |
| **Smoking Status, N (%)** |  |  | < 0.001 |
| Never smoker | 2140/4244 (50.4) | 1146/2701 (42.4) |  |
| Ex-smoker | 1515/4244 (35.7) | 1165/2701 (43.1) |  |
| Current smoker | 589/4244 (13.9) | 390/2701 (14.4) |  |
| **Drinking, N (%)** |  |  | < 0.001 |
| Current not drinking | 840/4503 (18.7) | 538/2854 (18.9) |  |
| Current moderate (1-14 unit/w) | 2689/4503 (59.7) | 1489/2854 (52.2) |  |
| Current heavy (>14 unit/w) | 974/4503 (21.6) | 827/2854 (29.0) |  |
| **Fruit/vegetable consumption, N (%)** |  |  | < 0.001 |
| < Daily | 1599/4501 (35.5) | 1241/2858 (43.4) |  |
| ≥ Daily | 2902/4501 (64.5) | 1617/2858 (56.6) |  |
| **Physical activity** |  |  | < 0.001 |
| Inactive | 826/4504 (18.3) | 641/2860 (22.4) |  |
| Moderate | 1620/4504 (36.0) | 1029/2860 (36.0) |  |
| Active | 2058/4504 (45.7) | 1190/2860 (41.6) |  |
| **Education level, N (%)** |  |  | < 0.001 |
| Low | 161/3382 (4.8) | 123/2237 (5.5) |  |
| Middle | 1672/3382 (49.4) | 1237/2237 (55.3) |  |
| High | 1549/3382 (45.8) | 877/2237 (39.2) |  |
| **Socioeconomic position, N (%)** |  |  | 0.016 |
| Low | 672/4503 (14.9) | 471/2856 (16.5) |  |
| Intermediate | 1813/4503 (40.3) | 1059/2856 (37.3) |  |
| High | 2018/4503 (44.8) | 1326/2856 (46.4) |  |

^a^ T-test or Wilcoxon rank-sum test for continuous variables and chi-square test for categorical variables;

^b^ Not follow normal distribution, shown with median (25th–75th percentiles);

WHtR, waist-to-height ratio; SD, standard deviation; IQR, interquartile range; CVD, cardiovascular disease; MI, myocardial infarction; TC, total cholesterol; TG, triglycerides; LDL-C, low-density lipoprotein cholesterol; HDL-C, high-density lipoprotein cholesterol; SBP, systolic blood pressure; DBP, diastolic blood pressure; BP, blood pressure; CRP, C-Reactive Protein; IL-6, Interleukin 6

**Supplementary Table S3d** Characteristics of the Sample II in Phase 3 *according to WHT.5R criterion*

| **Characteristic** | WHT.5R **<**  **75th population-wide centile**, **N (%)**  **N = 5666 (75.7)** | **WHT.5R ≥**  **75th population-wide centile N (%)**  **N = 1822 (24.3)** | **P value ^a^** |
| --- | --- | --- | --- |
| **Age, mean ± SD, yrs** | 49.66 ± 6.01 | 50.84 ± 5.92 | < 0.001 |
| **Sex (Female), N (%)** | 1984/5666 (35.0) | 311/1822 (17.1) | < 0.001 |
| **Ethnicity (White), N (%)** | 5178/5648 (91.7) | 1649/1820 (90.6) | 0.169 |
| **Height, mean ± SD, cm** | 171.52 ± 9.52 | 173.55 ± 8.87 | 0.072 |
| **Weight, mean ± SD, kg** | 70.48 ± 9.87 | 88..36 ± 10.72 | < 0.001 |
| **SBP, mean ± SD, mm Hg** | 118.71 ± 13.22 | 125.95 ± 13.04 | < 0.001 |
| **DBP, mean ± SD, mm Hg** | 78.16 ± 9.02 | 84.30 ± 8.95 | < 0.001 |
| **TC, mean ± SD, mmol/L** | 6.40 ± 1.14 | 6.72 ± 1.16 | < 0.001 |
| **TG, mean ± SD, mmol/L** | 1.28 ± 0.89 | 2.03 ± 1.42 | < 0.001 |
| **LDL-C, mean ± SD, mmol/L** | 4.32 ± 1.03 | 4.60 ± 1.02 | < 0.001 |
| **HDL-C, mean ± SD, mmol/L** | 1.50 ± 0.41 | 1.23 ± 0.33 | < 0.001 |
| **Fasting glucose, mean ± SD, mmol/L** | 5.16 ± 0.46 | 5.31 ± 0.47 | < 0.001 |
| **CRP, ^b^ median (IQR), mg/L** | 0.72 (0.38, 1.42) | 1.56 (0.84, 3.12) | < 0.001 |
| **IL-6, ^b^ median (IQR), pg/mL** | 1.31 (0.96, 1.88) | 1.73 (1.24, 2.57) | < 0.001 |
| **Baseline CVD diagnosis, N (%)** | 445/5666 (7.9) | 237/1822 (13.0) | < 0.001 |
| **Family history of CVD**  **(angina, MI, stroke), N (%)** | 2575/5003 (51.5) | 875/1600 (54.7) | 0.027 |
| **Family history of diabetes, N (%)** | 554/5586 (9.9) | 245/1786 (13.7) | < 0.001 |
| **Family history of hypertension, N (%)** | 2180/4894 (44.5) | 711/1533 (46.4) | 0.218 |
| **Smoking Status, N (%)** |  |  | < 0.001 |
| Never smoker | 2618/5257 (49.8) | 668/1688 (39.6) |  |
| Ex-smoker | 1916/5257 (36.4) | 764/1688 (45.3) |  |
| Current smoker | 723/5257 (13.8) | 256/1688 (15.2) |  |
| **Drinking, N (%)** |  |  | < 0.001 |
| Current not drinking | 1083/5584 (19.4) | 295/1773 (16.6) |  |
| Current moderate (1-14 unit/w) | 3262/5584 (58.4) | 916/1773 (51.7) |  |
| Current heavy (>14 unit/w) | 1239/5584 (22.2) | 562/1773 (31.7) |  |
| **Fruit/vegetable consumption, N (%)** |  |  | < 0.001 |
| < Daily | 2056/5584 (36.8) | 784/1775 (44.2) |  |
| ≥ Daily | 3528/5584 (63.2) | 991/1775 (55.8) |  |
| **Physical activity** |  |  | 0.007 |
| Inactive | 1084/5587 (19.4) | 383/1777 (21.6) |  |
| Moderate | 1983/5587 (35.5) | 666/1777 (37.5) |  |
| Active | 2520/5587 (45.1) | 728/1777 (41.0) |  |
| **Education level, N (%)** |  |  | 0.060 |
| Low | 211/4227 (5.0) | 73/1392 (5.2) |  |
| Middle | 2153/4227 (50.9) | 756/1392 (54.3) |  |
| High | 1863/4227 (44.1) | 563/1392 (40.4) |  |
| **Socioeconomic position, N (%)** |  |  | 0.162 |
| Low | 892/5586 (16.0) | 251/1773 (14.2) |  |
| Intermediate | 2160/5586 (38.7) | 712/1773 (40.2) |  |
| High | 2534/5586 (45.4) | 810/1773 (45.7) |  |

^a^ T-test or Wilcoxon rank-sum test for continuous variables and chi-square test for categorical variables;

^b^ Not follow normal distribution, shown with median (25th–75th percentiles);

WHT.5R, waist-by-height^0.5^; WHT.5R: 75th population-wide centile (6.911 cm^0.5^); SD, standard deviation; IQR, interquartile range; CVD, cardiovascular disease; MI, myocardial infarction; TC, total cholesterol; TG, triglycerides; LDL-C, low-density lipoprotein cholesterol; HDL-C, high-density lipoprotein cholesterol; SBP, systolic blood pressure; DBP, diastolic blood pressure; BP, blood pressure; CRP, C-Reactive Protein; IL-6, Interleukin 6

**Supplementary Table S3e** Characteristics of the Sample II in Phase 3 *according to ABSI criterion*

| **Characteristic** | ABSI **<**  **75th population-wide centile**, **N (%)**  **N = 5655 (75.5)** | **ABSI ≥**  **75th population-wide centile N (%)**  **N = 1833 (24.5)** | **P value ^a^** |
| --- | --- | --- | --- |
| **Age, mean ± SD, yrs** | 49.54 ± 5.97 | 51.21 ± 5.97 | < 0.001 |
| **Sex (Female), N (%)** | 2239/5655 (39.6) | 56/1833 (3.1) | < 0.001 |
| **Ethnicity (White), N (%)** | 5165/5640 (91.6) | 1662/1828 (90.9) | 0.169 |
| **Height, mean ± SD, cm** | 170.71 ± 9.59 | 176.02 ± 7.49 | < 0.001 |
| **Weight, mean ± SD, kg** | 72.85 ± 12.25 | 80.92 ± 11.97 | < 0.001 |
| **SBP, mean ± SD, mm Hg** | 119.38 ± 13.38 | 123.82 ± 13.45 | < 0.001 |
| **DBP, mean ± SD, mm Hg** | 78.62 ± 9.21 | 82.86 ± 9.18 | < 0.001 |
| **TC, mean ± SD, mmol/L** | 6.43 ± 1.16 | 6.64 ± 1.13 | < 0.001 |
| **TG, mean ± SD, mmol/L** | 1.33 ± 0.93 | 1.86 ± 1.43 | < 0.001 |
| **LDL-C, mean ± SD, mmol/L** | 4.33 ± 1.04 | 4.56 ± 0.99 | < 0.001 |
| **HDL-C, mean ± SD, mmol/L** | 1.49 ± 0.42 | 1.26 ± 0.33 | < 0.001 |
| **Fasting glucose, mean ± SD, mmol/L** | 5.17 ± 0.46 | 5.28 ± 0.47 | < 0.001 |
| **CRP, ^b^ median (IQR), mg/L** | 0.78 (0.40, 1.62) | 1.19 (0.63, 2.41) | < 0.001 |
| **IL-6, ^b^ median (IQR), pg/mL** | 1.34 (0.98, 1.98) | 1.55 (1.17, 2.25) | < 0.001 |
| **Baseline CVD diagnosis, N (%)** | 467/5655 (8.3) | 215/1833 (11.7) | < 0.001 |
| **Family history of CVD**  **(angina, MI, stroke), N (%)** | 2569/5005 (51.3) | 881/1598 (55.1) | 0.009 |
| **Family history of diabetes, N (%)** | 587/5574 (10.5) | 212/1798 (11.8) | 0.147 |
| **Family history of hypertension, N (%)** | 2201/4904 (44.9) | 690/1523 (45.3) | 0.794 |
| **Smoking Status, N (%)** |  |  | < 0.001 |
| Never smoker | 2574/5229 (49.2) | 712/1716 (41.5) |  |
| Ex-smoker | 1931/5229 (36.9) | 749/1716 (43.6) |  |
| Current smoker | 724/5229 (13.8) | 255/1716 (14.9) |  |
| **Drinking, N (%)** |  |  | < 0.001 |
| Current not drinking | 1111/5556 (20.0) | 267/1801 (14.8) |  |
| Current moderate (1-14 unit/w) | 3241/5556 (58.3) | 937/1801 (52.0) |  |
| Current heavy (>14 unit/w) | 1204/5556 (21.7) | 597/1801 (33.1) |  |
| **Fruit/vegetable consumption, N (%)** |  |  | < 0.001 |
| < Daily | 2017/5555 (36.3) | 823/1804 (45.6) |  |
| ≥ Daily | 3538/5555 (63.7) | 981/1804 (54.4) |  |
| **Physical activity** |  |  | 0.002 |
| Inactive | 1129/5560 (20.3) | 338/1804 (18.7) |  |
| Moderate | 1938/5560 (34.9) | 711/1804 (39.4) |  |
| Active | 2493/5560 (44.8) | 755/1804 (41.9) |  |
| **Education level, N (%)** |  |  | 0.125 |
| Low | 218/4251 (5.1) | 66/1368 (4.8) |  |
| Middle | 2168/4251 (51.0) | 741/1368 (54.2) |  |
| High | 1865/4251 (43.9) | 561/1368 (41.0) |  |
| **Socioeconomic position, N (%)** |  |  | < 0.001 |
| Low | 989/5558 (17.8) | 154/1801 (8.6) |  |
| Intermediate | 2074/5558 (37.3) | 798/1801 (44.3) |  |
| High | 2495/5558 (44.9) | 849/1801 (47.1) |  |

^a^ T-test or Wilcoxon rank-sum test for continuous variables and chi-square test for categorical variables;

^b^ Not follow normal distribution, shown with median (25th–75th percentiles);

ABSI, a body shape index; ABSI: 75th population-wide centile (0.078 m^7/6^/kg^2/3^); SD, standard deviation; IQR, interquartile range; CVD, cardiovascular disease; MI, myocardial infarction; TC, total cholesterol; TG, triglycerides; LDL-C, low-density lipoprotein cholesterol; HDL-C, high-density lipoprotein cholesterol; SBP, systolic blood pressure; DBP, diastolic blood pressure; BP, blood pressure; CRP, C-Reactive Protein; IL-6, Interleukin 6

**Supplementary Table S4** Characteristics of the *participants with complete data* in Phase 3 (baseline time)

| Characteristic | Participants with complete data of Sample I, N (%)  N = 7206 (100) | Participants **with complete data of Sample II, N (%)**  **N = 6813 (100)** |
| --- | --- | --- |
| Baseline Diabetes, N (%) | 228/7206 (3.2) | 0/6813 (0) |
| Age, mean ± SD, yrs | 50.09 ± 6.04 | 49.97 ± 6.02 |
| < 50 | 3789/7206 (52.6) | 3640/6813 (53.4) |
| ≥ 50 | 3417/7206 (47.4) | 3173/6813 (46.6) |
| Sex, N (%) |  |  |
| Male | 5013/7206 (69.6) | 4753/6813 (69.8) |
| Female | 2193/7206 (30.4) | 2060/6813 (30.2) |
| Ethnicity, N (%) |  |  |
| White | 6564/7206 (91.1) | 6266/6813 (92.0) |
| Non-white | 642/7206 (8.9) | 547/6813 (8.0) |
| Height, mean ± SD, cm | 171.95 ± 9.35 | 172.07 ± 9.32 |
| Weight, mean ± SD, kg | 74.81 ± 12.67 | 74.77 ± 12.61 |
| BMI, mean ± SD, kg/m^2^ | 25.27 ± 3.71 | 25.22 ± 3.67 |
| WC, mean ± SD, cm | 83.75 ± 11.48 | 83.60 ± 11.41 |
| WHtR, mean ± SD | 0.49 ± 0.06 | 0.49 ± 0.06 |
| WHT.5R, mean ± SD, cm^0.5^ | 6.38 ± 0.83 | 6.37 ± 0.82 |
| ABSI, mean ± SD, m^7/6^/kg^2/3^ | 0.07 ± 0.01 | 0.07 ± 0.01 |
| SBP, mean ± SD, mm Hg | 120.66 ± 13.64 | 120.45 ± 13.54 |
| DBP, mean ± SD, mm Hg | 79.75 ± 9.38 | 79.65 ± 9.35 |
| TC, mean ± SD, mmol/L | 6.49 ± 1.15 | 6.48 ± 1.16 |
| TG, mean ± SD, mmol/L | 1.48 ± 1.12 | 1.46 ± 1.09 |
| LDL-C, mean ± SD, mmol/L | 4.39 ± 1.03 | 4.39 ± 1.04 |
| HDL-C, mean ± SD, mmol/L | 1.43 ± 0.41 | 1.44 ± 0.41 |
| Fasting glucose, mean ± SD, mmol/L | 5.24 ± 0.69 | 5.19 ± 0.47 |
| CRP, ^a^ median (IQR), mg/L | 0.89 (0.45, 1.91) | 0.87 (0.44, 1.85) |
| IL-6, ^a^ median (IQR), pg/mL | 1.41 (1.03, 2.07) | 1.39 (1.02, 2.03) |
| Baseline CVD diagnosis, N (%) |  |  |
| Yes | 688/7206 (9.5) | 6178/6813 (90.7) |
| **No** | 6518/7206 (90.5) | 635/6813 (9.3) |
| **Family history of CVD**  **(angina, MI, stroke), N (%)** | 3327/6394 (52.0) | 3151/6043 (52.1) |
| **Family history of diabetes, N (%)** | 828/7206 (11.5) | 742/6813 (10.9) |
| **Family history of hypertension, N (%)** | 2774/6229 (44.5) | 2633/5888 (44.7) |
| **Smoking Status, N (%)** |  |  |
| Never smoker | 3389/7206 (47.0) | 3226/6813 (47.4) |
| Ex-smoker | 2774/7206 (38.5) | 2623/6813 (38.5) |
| Current smoker | 1043/7206 (14.5) | 964/6813 (14.1) |
| **Drinking, N (%)** |  |  |
| Current not drinking | 1370/7206 (19.0) | 1253/6813 (18.4) |
| Current moderate (1-14 unit/w) | 4058/7206 (56.3) | 3864/6813 (57.7) |
| Current heavy (>14 unit/w) | 1778/7206 (24.7) | 1696/6813 (24.9) |
| **Fruit/vegetable consumption, N (%)** |  |  |
| < Daily | 2777/7206 (38.5) | 2604/6813 (38.2) |
| ≥ Daily | 4429/7206 (61.5) | 4209/6813 (61.8) |
| **Physical activity** |  |  |
| Inactive | 1451/7206 | 1326/6813 (19.5) |
| Moderate | 2568/7206 | 2450/6813 (36.0) |
| Active | 3187/7206 | 3037/6813 (44.6) |
| **Education level, N (%)** |  |  |
| Low | 263/5444 (4.8) | 249/5147 (4.8) |
| Middle | 2857/5444 (52.5) | 2687/5147 (52.2) |
| High | 2324/5444 (42.7) | 2211/5147 (43.0) |
| **Socioeconomic position, N (%)** |  |  |
| Low | 1159/7206 (16.1) | 1023/6813 (15.0) |
| Intermediate | 2757/7206 (38.2) | 2670/6813 (38.2) |
| High | 3290/7206 (45.7) | 3120/6813 (45.8) |

^a^ Not follow normal distribution, shown with median (25th–75th percentiles);

SD, standard deviation; IQR, interquartile range; BMI, body mass index; WC, waist circumference; WHtR, waist-to-height ratio; WHT.5R, waist-by-height^0.5^; ABSI, a body shape index; CVD, cardiovascular disease; MI, myocardial infarction; TC, total cholesterol; TG, triglycerides; LDL-C, low-density lipoprotein cholesterol; HDL-C, high-density lipoprotein cholesterol; SBP, systolic blood pressure; DBP, diastolic blood pressure; CRP, C-Reactive Protein; IL-6, Interleukin 6

**Supplementary Table S5a** Characteristics of the Sample I in Phase 3 according to sex

| **Characteristic** | Male, **N (%)**  **N = 5511 (69.1)** | **Female, N (%)**  **N = 2468 (30.9)** | **P value ^a^** |
| --- | --- | --- | --- |
| **Baseline diabetes** | 183/5511 (3.3) | 88/2468 (3.6) | 0.623 |
| **Age, mean**±**SD, yrs** | 49.75 ± 5.98 | 50.76 ± 6.08 | < 0.001 |
| **Ethnicity (White), N (%)** | 5098/5500 (92.7) | 2090/2451 (85.3) | < 0.001 |
| **Height, mean**±**SD, cm** | 176.36 ± 6.71 | 161.81 ± 6.49 | < 0.001 |
| **Weight, mean**±**SD, kg** | 78.27 ± 11.21 | 67.33 ± 12.72 | < 0.001 |
| **BMI, mean**±**SD, kg/m^2^** | 25.14 ± 3.18 | 25.72 ± 4.73 | < 0.001 |
| **WC, mean**±**SD, cm** | 87.47 ± 9.31 | 75.68 ± 11.73 | < 0.001 |
| **WHtR, mean**±**SD** | 0.50 ± 0.05 | 0.47 ± 0.07 | < 0.001 |
| **WHT.5R, mean**±**SD, cm^0.5^** | 6.59 ± 0.70 | 5.95 ± 0.93 | < 0.001 |
| **ABSI, mean**±**SD, m^7/6^/kg^2/3^** | 0.08 ± 0.00 | 0.07 ± 0.01 | < 0.001 |
| SBP, mean±SD, mm Hg | 122.00 ± 13.23 | 117.78 ± 14.05 | < 0.001 |
| DBP, mean±SD, mm Hg | 81.10 ± 9.19 | 76.75 ± 9.19 | < 0.001 |
| TC, mean±SD, mmol/L | 6.47 ± 1.13 | 6.51 ± 1.21 | 0.161 |
| TG, mean±SD, mmol/L | 1.61 ± 1.23 | 1.21 ± 0.78 | <0.001 |
| LDL-C, mean±SD, mmol/L | 4.44 ± 0.99 | 4.29 ± 1.11 | <0.001 |
| HDL-C, mean±SD, mmol/L | 1.32 ± 0.35 | 1.68 ± 0.43 | <0.001 |
| Fasting glucose, mean±SD, mmol/L | 5.31 ± 0.69 | 5.09 ± 0.69 | < 0.001 |
| CRP, ^b^ median (IQR), mg/L | 0.85 (0.43, 1.73) | 1.02 (0.47, 2.36) | <0.001 |
| IL-6, ^b^ median (IQR), pg/mL | 1.35 (1.00, 1.94) | 1.57 (1.11, 2.45) | < 0.001 |
| **Family history of diabetes, N (%)** | 571/5434 (10.5) | 335/2423 (13.8) | < 0.001 |
| **Family history of CVD**  **(angina, MI, stroke), N (%)** | 2519/4851 (51.9) | 1152/2188 (52.7) | 0.592 |
| **Smoking Status, N (%)** |  |  | < 0.001 |
| Never smoker | 2288/5103 (44.8) | 1164/2246 (51.8) |  |
| Ex-smoker | 2149/5103 (42.1) | 690/2246 (30.7) |  |
| Current smoker | 666/5103 (13.1) | 392/2246 (17.5) |  |
| **Drinking, N (%)** |  |  | < 0.001 |
| Current not drinking | 780/5395 (14.5) | 733/2410 (30.4) |  |
| Current moderate | 2946/5395 (54.6) | 1453/2410 (60.3) |  |
| Current heavy | 1669/5395 (30.9) | 224/2410 (9.3) |  |
| **Fruit/vegetable consumption, N (%)** |  |  | < 0.001 |
| < Daily | 2221/5396 (41.2) | 810/2411 (34.0) |  |
| ≥ Daily | 3175/5396 (58.8) | 1592/2411 (66.0) |  |
| **Education level, N (%)** |  |  | < 0.001 |
| Low | 201/4177 (4.8) | 99/1812 (5.5) |  |
| Middle | 2062/4177 (49.4) | 1058/1812 (58.4) |  |
| High | 1914/4177 (45.8) | 655/1812 (36.1) |  |
| **Physical activity** |  |  | <0.001 |
| Inactive | 767/5400 (14.2) | 843/2412 (35.0) |  |
| Moderate | 1975/5400 (36.6) | 805/2412 (33.4) |  |
| Active | 2658/5400 (49.2) | 764/2412 (31.7) |  |
| **Socioeconomic position, N (%)** |  |  | <0.001 |
| Low | 356/5395 (6.6) | 941/2411 (39.0) |  |
| Intermediate | 2590/5395 (48.0) | 383/2411 (15.9) |  |
| High | 2449/5395 (45.4) | 1087/2411 (45.1) |  |
| **Menopause, N (%)** | NA | 1252/2408 (52.0) | NA |

^a^ T-test or Wilcoxon rank-sum test for continuous variables and chi-square test for categorical variables;

^b^ not follow normal distribution, shown with median (25th-75th percentiles);

SD, standard deviation; IQR, interquartile range; BMI, body mass index; WC, waist circumference; WHtR, waist-to-height ratio; WHT.5R, waist-by-height^0.5^; ABSI, a body shape index; CVD, cardiovascular disease; MI, myocardial infarction; TC, total cholesterol; TG, triglycerides; LDL-C, low-density lipoprotein cholesterol; HDL-C, high-density lipoprotein cholesterol; SBP, systolic blood pressure; DBP, diastolic blood pressure; CRP, C-Reactive Protein; IL-6, Interleukin 6

**Supplementary Table S5b** Characteristics of the Sample II in Phase 3 according to sex

| **Characteristic** | Male, **N (%)**  **N = 5193 (69.4)** | **Female, N (%)**  **N = 2295 (30.6)** | **P value** ^a^ |
| --- | --- | --- | --- |
| **Age, mean ± SD, yrs** | 49.66 ± 5.96 | 50.60 ± 6.07 | < 0.001 |
| **Ethnicity (White), N (%)** | 4855/5185 (93.6) | 1972/2283 (86.4) | < 0.001 |
| **Height, mean ± SD, cm** | 176.46 ± 6.67 | 161.95 ± 6.48 | < 0.001 |
| **Weight, mean ± SD, kg** | 78.22 ± 11.10 | 67.16 ± 12.66 | < 0.001 |
| **BMI, mean ± SD, kg/m^2^** | 25.10 ± 3.13 | 25.62 ± 4.68 | < 0.001 |
| **WC, mean ± SD, cm** | 87.32 ± 9.22 | 75.34 ± 11.55 | < 0.001 |
| **WHtR, mean ± SD** | 0.50 ± 0.05 | 0.47 ± 0.07 | < 0.001 |
| **WHT.5R, mean ± SD, cm^0.5^** | 6.58 ± 0.69 | 5.92 ± 0.91 | < 0.001 |
| **ABSI, mean ± SD, m^7/6^/kg^2/3^** | 0.08 ± 0.00 | 0.07 ± 0.01 | < 0.001 |
| **SBP, mean ± SD, mm Hg** | 121.73 ± 13.14 | 117.62 ± 13.97 | < 0.001 |
| **DBP, mean ± SD, mm Hg** | 80.96 ± 9.16 | 76.72 ± 9.22 | < 0.001 |
| TC, mean ± SD, mmol/L | 6.47 ± 1.13 | 6.50 ± 1.21 | 0.232 |
| TG, mean ± SD, mmol/L | 1.58 ± 1.20 | 1.19 ± 0.76 | < 0.001 |
| LDL-C, mean ± SD, mmol/L | 4.44 ± 1.00 | 4.27 ± 1.11 | < 0.001 |
| HDL-C, mean ± SD, mmol/L | 1.32 ± 0.35 | 1.69 ± 0.43 | < 0.001 |
| Fasting glucose, mean ± SD, mmol/L | 5.26 ± 0.45 | 5.05 ± 0.47 | < 0.001 |
| CRP, ^b^ median (IQR), mg/L | 0.83 (0.43, 1.69) | 1.00 (0.46, 2.23) | < 0.001 |
| IL-6, ^b^ median (IQR), pg/mL | 1.34 (0.98, 1.91) | 1.55 (1.10, 2.41) | < 0.001 |
| **Family history of diabetes, N (%)** | 501/5120 (9.8) | 298/2252 (13.2) | < 0.001 |
| **Family history of CVD**  **(angina, MI, stroke), N (%)** | 2379/4574 (52.0) | 1071/2029 (52.8) | 0.580 |
| **Smoking Status, N (%)** |  |  | < 0.001 |
| Never smoker | 2191/4837 (45.3) | 1095/2108 (51.9) |  |
| Ex-smoker | 2031/4837 (42.0) | 649/2108 (30.8) |  |
| Current smoker | 615/4837 (12.7) | 364/2108 (17.3) |  |
| **Drinking, N (%)** |  |  | < 0.001 |
| Current not drinking | 716/5102 (14.0) | 662/2255 (29.4) |  |
| Current moderate (1-14 unit/w) | 2801/5102 (54.9) | 1377/2255 (61.1) |  |
| Current heavy (>14 unit/w) | 1585/5102 (31.1) | 216/2255 (9.6) |  |
| **Fruit/vegetable consumption, N (%)** |  |  | < 0.001 |
| < Daily | 2084/5103 (40.8) | 756/2256 (33.5) |  |
| ≥ Daily | 3019/5103 (59.2) | 1500/2256 (66.5) |  |
| **Physical activity** |  |  | <0.001 |
| Inactive | 692/5107 (13.6) | 775/2257 (34.3) |  |
| Moderate | 1885/5107 (36.9) | 764/2257 (33.9) |  |
| Active | 2530/5107 (49.5) | 718/2257 (31.8) |  |
| **Socioeconomic position, N (%)** |  |  | <0.001 |
| Low | 300/5103 (5.9) | 843/2256 (37.4) |  |
| Intermediate | 2497/5103 (48.9) | 375/2256 (16.6) |  |
| High | 2306/5103 (45.2) | 1038/2256 (46.0) |  |
| **Menopause, N (%)** | NA | 1146/2253 (50.9) | NA |

^a^ T-test or Wilcoxon rank-sum test for continuous variables and chi-square test for categorical variables;

^b^ Not follow normal distribution, shown with median (25th-75th percentiles);

SD, standard deviation; IQR, interquartile range; BMI, body mass index; WC, waist circumference; WHtR, waist-to-height ratio; WHT.5R, waist-by-height^0.5^; ABSI, a body shape index; CVD, cardiovascular disease; MI, myocardial infarction; TC, total cholesterol; TG, triglycerides; LDL-C, low-density lipoprotein cholesterol; HDL-C, high-density lipoprotein cholesterol; SBP, systolic blood pressure; DBP, diastolic blood pressure; CRP, C-Reactive Protein; IL-6, Interleukin 6

**Supplementary Table S6** Correlation matrix for correlation between different adiposity indicators among Sample I, Sample II, and the participants with complete data from the Sample I and Sample II

1. Sample I (N=7979)

|  | **Weight** | **Height** | **BMI** | **WC** | **WHtR** | **WHT.5R** | **ABSI** |
| --- | --- | --- | --- | --- | --- | --- | --- |
| **Weight** | - |  |  |  |  |  |  |
| **Height** | 0.55 | - |  |  |  |  |  |
| **BMI** | 0.76 | -0.11 | - |  |  |  |  |
| **WC** | 0.87 | 0.39 | 0.73 | - |  |  |  |
| **WHtR** | 0.71 | -0.01 | 0.85 | 0.92 | - |  |  |
| **WHT.5R** | 0.81 | 0.21 | 0.81 | 0.98 | 0.98 | - |  |
| **ABSI** | 0.41 | 0.49 | 0.12 | 0.74 | 0.59 | 0.68 | - |

1. Sample II (N=7488)

|  | **Weight** | **Height** | **BMI** | **WC** | **WHtR** | **WHT.5R** | **ABSI** |
| --- | --- | --- | --- | --- | --- | --- | --- |
| **Weight** | - |  |  |  |  |  |  |
| **Height** | 0.55 | - |  |  |  |  |  |
| **BMI** | 0.76 | -0.11 | - |  |  |  |  |
| **WC** | 0.87 | 0.40 | 0.73 | - |  |  |  |
| **WHtR** | 0.71 | 0.01 | 0.85 | 0.92 | - |  |  |
| **WHT.5R** | 0.81 | 0.22 | 0.80 | 0.98 | 0.98 | - |  |
| **ABSI** | 0.42 | 0.49 | 0.12 | 0.74 | 0.59 | 0.68 | - |

1. Participants with *complete data* among the *Sample I* (N=7206)

|  | **Weight** | **Height** | **BMI** | **WC** | **WHtR** | **WHT.5R** | **ABSI** |
| --- | --- | --- | --- | --- | --- | --- | --- |
| **Weight** | - |  |  |  |  |  |  |
| **Height** | 0.55 | - |  |  |  |  |  |
| **BMI** | 0.77 | -0.11 | - |  |  |  |  |
| **WC** | 0.87 | 0.40 | 0.73 | - |  |  |  |
| **WHtR** | 0.71 | 0.00 | 0.85 | 0.92 | - |  |  |
| **WHT.5R** | 0.81 | 0.21 | 0.80 | 0.98 | 0.98 | - |  |
| **ABSI** | 0.42 | 0.49 | 0.12 | 0.74 | 0.59 | 0.68 | - |

1. Participants with *complete data* among the *Sample II* (N=6813)

|  | **Weight** | **Height** | **BMI** | **WC** | **WHtR** | **WHT.5R** | **ABSI** |
| --- | --- | --- | --- | --- | --- | --- | --- |
| **Weight** | - |  |  |  |  |  |  |
| **Height** | 0.55 | - |  |  |  |  |  |
| **BMI** | 0.77 | -0.10 | - |  |  |  |  |
| **WC** | 0.87 | 0.41 | 0.73 | - |  |  |  |
| **WHtR** | 0.71 | 0.02 | 0.84 | 0.92 | - |  |  |
| **WHT.5R** | 0.81 | 0.22 | 0.80 | 0.98 | 0.98 | - |  |
| **ABSI** | 0.42 | 0.50 | 0.12 | 0.74 | 0.60 | 0.69 | - |

BMI, body mass index; WC, waist circumference; WHtR, waist-to-height ratio; WHT.5R, waist-by-height^0.5^; ABSI, a body shape index

**Supplementary Table S7** Area under the receiver operating characteristic curve for five adiposity indicators in relation to diabetes in the participants with complete data of the Sample I

| **Exposures** | **AUC_3_ (95% CI)**  **Complete data of Sample I:**  **no covariates** | **AUC_4_ (95% CI)**  **Complete data of Sample I:**  **with covariates** |
| --- | --- | --- |
| **BMI** | 0.575 (0.536, 0.614) | 0.714 (0.677, 0.750) |
| **WC** | 0.602 (0.563, 0.641) | 0.715 (0.678, 0.752) |
| **WHtR** | 0.629 (0.590, 0.668) | 0.718 (0.682, 0.755) |
| **WHT.5R** | 0.618 (0.579, 0.657) | 0.717 (0.680, 0.754) |
| **ABSI** | 0.614 (0.576, 0.653) | 0.720 (0.684, 0.757) |

AUC_3_, in the participants with complete data among Sample I (sample size=7,206, number of events =228), without covariates;

AUC_4_, in the participants with complete data among Sample I (sample size=7,206, number of events =228), including covariates for age, sex, ethnicity, smoking, drinking, socioeconomic position, physical activity, dietary behavior, family history of diabetes, baseline CVD diagnosis, and CVD medication.

AUC, area under the curve; CVD, cardiovascular disease; CI, confidence interval; BMI, body mass index; WC, waist circumference; WHtR, waist-to-height ratio; WHT.5R, waist-by-height^0.5^; ABSI, a body shape index.

**Supplementary Table S8** Area under the receiver operating characteristic curve for five adiposity indices in identifying diabetes in Sample I stratified by sex

| **Exposures** | **AUC (95% CI)**  **Male:**  **with covariates** | **AUC (95% CI)**  **Female:**  **with covariates** |
| --- | --- | --- |
| **BMI** | 0.734 (0.730, 0.738) | 0.701 (0.695, 0.707) |
| **WC** | 0.736 (0.732, 0.740) | 0.708 (0.702, 0.714) |
| **WHtR** | 0.739 (0.735, 0.743) | 0.712 (0.706, 0.718) |
| **WHT.5R** | 0.738 (0.734, 0.742) | 0.710 (0.704, 0.716) |
| **ABSI** | 0.738 (0.734, 0.742) | 0.712 (0.706, 0.718) |

AUC, among Sample I (sample size=7,979, number of events=271), including covariates for age, ethnicity, smoking, drinking, socioeconomic position, physical activity, dietary behavior, family history of diabetes, baseline CVD diagnosis, CVD medication, menopause status (females subjects only), and imputation for the missing covariates;

AUC, area under the curve; CVD, cardiovascular disease; CI, confidence interval; BMI, body mass index; WC, waist circumference; WHtR, waist-to-height ratio; WHT.5R, waist-by-height^0.5^; ABSI, a body shape index

**Supplementary Table S9** Estimated hazard ratios (Per 1-SD Increase) for incident diabetes in relation to the five adiposity indices (continuous)

|  | **Adjusted HR_2_,** ^a^ **(95% CI)** | **Adjusted HR_2_ with MI, ^b^ (95% CI)** |
| --- | --- | --- |
| Standardized BMI | 1.56 (1.48, 1.65) | 1.59 (1.51, 1.67) |
| Standardized WC | 1.80 (1.68, 1.93) | 1.83 (1.72, 1.96) |
| Standardized WHtR | 1.75 (1.65, 1.86) | 1.78 (1.68, 1.89) |
| Standardized WHT.5R | 1.78 (1.67, 1.90) | 1.81 (1.70, 1.93) |
| Standardized ABSI | 1.67 (1.51, 1.84) | 1.68 (1.52, 1.84) |

^a^ Using complete data (n=6,813) of Sample II, number of events for complete data=847;

^b^ Using Sample II, sample size=7,488, number of events=940, imputation for the missing covariates;

Adjusted HR_2_, the Cox regression model was adjusted for age, sex, ethnicity, smoking, drinking, socioeconomic position, physical activity, dietary behavior, family history of diabetes, baseline CVD diagnosis, and CVD medication;

Adjusted HR_2_ with MI, the imputed Cox regression model was adjusted for age, sex, ethnicity, smoking, drinking, socioeconomic position, physical activity, dietary behavior, family history of diabetes, baseline CVD diagnosis, and CVD medication;

MI, multivariate imputation; CVD, cardiovascular disease; HR, hazard ratio; CI, confidence interval; BMI, body mass index; WC, waist circumference; WHtR, waist-to-height ratio; WHT.5R, waist-by-height^0.5^; ABSI, a body shape index.

Supplementary Table S10 Cumulative event (diabetes) rates for different subgroups at the end of follow-up

| **Subgroup** | **Participants with complete data of Sample II** | | | **Sample II** | | | |
| --- | --- | --- | --- | --- | --- | --- | --- |
|  | **N (%)** | **Diabetes, N (%)** | **P value** | **N (%)** | **Diabetes,**  **N (%)** | **Median follow-up time, yrs** | **P value** |
| **Sex** |  |  | 0.489 |  |  |  | 0.311 |
| Male | 4753 (69.8) | 582 (12.2) |  | 5193 (69.4) | 638 (12.3) | 16.1 |  |
| Female | 2057 (30.2) | 265 (12.9) |  | 2295 (30.6) | 302(13.2) | 16.0 |  |
| **Age, yrs** |  |  | <0.001 |  |  |  | <0.001 |
| < 50 | 3640 (53.4) | 374 (10.3) |  | 4035 (53.9) | 420 (10.4) | 16.1 |  |
| ≥50 | 3173 (46.6) | 473 (14.9) |  | 3453 (46.1) | 520 (15.1) | 16.0 |  |
| **Ethnicity** |  |  | <0.001 |  |  |  | <0.001 |
| White | 6266 (92.0) | 697 (11.1) |  | 6827 (91.4) | 767 (11.2) | 16.1 |  |
| Non-white | 547 (8.0) | 150 (27.4) |  | 641 (8.6) | 172 (26.8) | 15.3 |  |
| **Baseline CVD diagnosis** |  |  | 0.002 |  |  |  | 0.004 |
| No | 6178 (90.7) | 743 (12.0) |  | 6806 (90.9) | 830 (12.2) | 16.1 |  |
| Yes | 635 (9.3) | 104 (16.4) |  | 682 (9.1) | 110 (16.1) | 16.0 |  |

CVD, cardiovascular disease; P value for the differences in number of event (diabetes) between different subgroups

Supplementary Table S11 Subgroup analyses for incident diabetes in relation to high-value group by different adiposity indicators in cohort part

| **Stratification** | **HR, ^a^ (95% CI)** | **P value ^a^** | **P value for interaction ^a^** | **HR, ^b^ (95% CI)** | **P value ^b^** | **P value for interaction ^b^** |
| --- | --- | --- | --- | --- | --- | --- |
| **Sex** | | | |  |  |  |
| **BMI** |  |  | 0.148 |  |  | 0.119 |
| Male | 2.11 (1.77, 2.50) | < 0.001 |  | 2.03 (1.72, 2.40) | < 0.001 |  |
| Female | 2.56 (1.94, 3.38) | < 0.001 |  | 2.44 (1.88, 3.17) | < 0.001 |  |
| **WC** |  |  | 0.118 |  |  | 0.053 |
| Male | 2.22 (1.87, 2.62) | < 0.001 |  | 2.19 (1.87, 2.57) | < 0.001 |  |
| Female | 2.72 (2.11, 3.51) | < 0.001 |  | 2.76 (2.17, 3.50) | < 0.001 |  |
| **WHtR** |  |  | 0.152 |  |  | 0.061 |
| Male | 2.52 (2.10, 3.01) | < 0.001 |  | 2.45 (2.06, 2.90) | < 0.001 |  |
| Female | 3.02 (2.33, 3.91) | < 0.001 |  | 3.02 (2.37, 3.86) | < 0.001 |  |
| **WHT.5R** |  |  | 0.018 |  |  | 0.001 |
| Male | 2.32 (1.97, 2.75) | < 0.001 |  | 2.33 (1.98, 2.73) | < 0.001 |  |
| Female | 3.23 (2.48, 4.22) | < 0.001 |  | 3.50 (2.73, 4.49) | < 0.001 |  |
| **ABSI** |  |  | 0.997 |  |  | 0.719 |
| Male | 1.75 (1.47, 2.07) | < 0.001 |  | 1.71 (1.45, 2.01) | < 0.001 |  |
| Female | 1.48 (0.84, 2.62) | 0.178 |  | 1.62 (0.97, 2.72) | 0.068 |  |
| **Age, yrs** | | | | | | |
| **BMI** |  |  | 0.061 |  |  | 0.039 |
| < 50 | 2.71 (2.17, 3.38) | < 0.001 |  | 2.51 (2.03, 3.10) | < 0.001 |  |
| ≥ 50 | 1.92 (1.58, 2.33) | < 0.001 |  | 1.91 (1.59, 2.30) | < 0.001 |  |
| **WC** |  |  | 0.005 |  |  | 0.011 |
| < 50 | 2.90 (2.35, 3.58) | < 0.001 |  | 2.79 (2.28, 3.40) | < 0.001 |  |
| ≥ 50 | 2.02 (1.68, 2.43) | < 0.001 |  | 2.06 (1.73, 2.46) | < 0.001 |  |
| **WHtR** |  |  | 0.001 |  |  | 0.002 |
| < 50 | 3.41 (2.74, 4.25) | < 0.001 |  | 3.23 (2.62, 3.97) | < 0.001 |  |
| ≥ 50 | 2.23 (1.83, 2.71) | < 0.001 |  | 2.25 (1.87, 2.71) | < 0.001 |  |
| **WHT.5R** |  |  | 0.004 |  |  | 0.011 |
| < 50 | 3.10 (2.49, 3.86) | < 0.001 |  | 3.05 (2.48, 3.75) | < 0.001 |  |
| ≥ 50 | 2.19 (1.81, 2.65) | < 0.001 |  | 2.32 (1.93, 2.78) | < 0.001 |  |
| **ABSI** |  |  | 0.033 |  |  | 0.015 |
| < 50 | 1.94 (1.52, 2.47) | < 0.001 |  | 1.90 (1.52, 2.39) | < 0.001 |  |
| ≥ 50 | 1.57 (1.27, 1.95) | < 0.001 |  | 1.55 (1.26, 1.91) | < 0.001 |  |
| **Ethnicity** | | | |  |  |  |
| **BMI** |  |  | 0.016 |  |  | 0.018 |
| White | 2.44 (2.07, 2.87) | < 0.001 |  | 2.34 (2.01, 2.74) | < 0.001 |  |
| Non-white | 1.48 (1.04, 2.11) | 0.028 |  | 1.49 (1.08, 2.08) | 0.017 |  |
| **WC** |  |  | 0.154 |  |  | 0.302 |
| White | 2.47 (2.11, 2.88) | < 0.001 |  | 2.43 (2.10, 2.82) | < 0.001 |  |
| Non-white | 1.97 (1.41, 2.75) | < 0.001 |  | 2.10 (1.53, 2.88) | < 0.001 |  |
| **WHtR** |  |  | 0.268 |  |  | 0.227 |
| White | 2.74 (2.33, 3.21) | < 0.001 |  | 2.71 (2.32, 3.16) | < 0.001 |  |
| Non-white | 2.42 (1.68, 3.47) | < 0.001 |  | 2.35 (1.68, 3.29) | < 0.001 |  |
| **WHT.5R** |  |  | 0.020 |  |  | 0.091 |
| White | 2.74 (2.34, 3.21) | < 0.001 |  | 2.76 (2.37, 3.21) | < 0.001 |  |
| Non-white | 1.86 (1.32, 2.63) | < 0.001 |  | 2.12 (1.53, 2.93) | < 0.001 |  |
| **ABSI** |  |  | 0.272 |  |  | 0.217 |
| White | 1.71 (1.44, 2.04) | < 0.001 |  | 1.69 (1.43, 2.00) | < 0.001 |  |
| Non-white | 1.71 (1.13, 2.59) | 0.011 |  | 1.60 (1.07, 2.38) | 0.021 |  |
| **Baseline CVD diagnosis** | | | |  |  |  |
| **BMI** |  |  | 0.067 |  |  | 0.115 |
| Without CVD | 2.12 (1.82, 2.47) | < 0.001 |  | 2.07 (1.79, 2.39) | < 0.001 |  |
| With CVD | 4.00 (2.40, 6.69) | < 0.001 |  | 3.49 (2.15, 5.68) | < 0.001 |  |
| **WC** |  |  | 0.087 |  |  | 0.129 |
| Without CVD | 2.23 (1.92, 2.59) | < 0.001 |  | 2.25 (1.96, 2.59) | < 0.001 |  |
| With CVD | 3.74 (2.40, 5.82) | < 0.001 |  | 3.55 (2.31, 5.46) | < 0.001 |  |
| **WHtR** |  |  | 0.535 |  |  | 0.684 |
| Without CVD | 2.60 (2.22, 3.04) | < 0.001 |  | 2.58 (2.23, 2.99) | < 0.001 |  |
| With CVD | 3.38 (2.11, 5.42) | < 0.001 |  | 3.23 (2.04, 5.10) | < 0.001 |  |
| **WHT.5R** |  |  | 0.107 |  |  | 0.123 |
| Without CVD | 2.60 (2.22, 3.04) | < 0.001 |  | 2.47 (2.14, 2.86) | < 0.001 |  |
| With CVD | 3.38 (2.11, 5.42) | < 0.001 |  | 3.98 (2.63, 6.01) | < 0.001 |  |
| **ABSI** |  |  | 0.223 |  |  | 0.205 |
| Without CVD | 1.74 (1.47, 2.07) | < 0.001 |  | 1.70 (1.44, 2.00) | < 0.001 |  |
| With CVD | 1.41 (0.89, 2.22) | 0.141 |  | 1.36 (0.87, 2.12) | 0.177 |  |

^a^ Using complete data (n=6,813) of Sample II, number of events for complete data=847;

^b^ Using Sample II, sample size=7,488, number of events =940, imputation for the missing covariates;

Sex stratification: controlling covariates for age, ethnicity, smoking, drinking, socioeconomic position, physical activity, dietary behavior, family history of diabetes, baseline CVD diagnosis, CVD medication, and menopause status (females subjects only).

Age stratification: controlling covariates for sex, ethnicity, smoking, drinking, socioeconomic position, physical activity, dietary behavior, family history of diabetes, baseline CVD diagnosis, and CVD medication.

Ethnicity stratification: controlling covariates for age, sex, smoking, drinking, socioeconomic position, physical activity, dietary behavior, family history of diabetes, baseline CVD diagnosis, and CVD medication.

Baseline CVD diagnosis stratification: controlling covariates for age, sex, ethnicity, smoking, drinking, socioeconomic position, physical activity, dietary behavior, family history of diabetes, and CVD medication.

CVD, cardiovascular disease; HR, hazard ratio; CI, confidence interval; BMI, body mass index; WC, waist circumference; WHtR, waist-to-height ratio; WHT.5R, waist-by-height^0.5^; ABSI, a body shape index

**Supplementary Table S12** Log-rank test p-values for pairwise comparisons among the different levels of BMI and WHtR combination

1. **Among Sample II**

|  | **low BMI and low WHtR** | **high BMI and low WHtR** | **low BMI and high WHtR** | **high BMI and high WHtR** |
| --- | --- | --- | --- | --- |
| **low BMI and low WHtR** | - |  |  |  |
| **high BMI and low WHtR** | < 0.001 | - |  |  |
| **low BMI and high WHtR** | <0.001 | <0.001 | - |  |
| **high BMI and high WHtR** | < 0.001 | <0.001 | 0.301 | - |

**b. Among the participants with complete data of Sample II**

|  | **low BMI and low WHtR** | **high BMI and low WHtR** | **low BMI and high WHtR** | **high BMI and high WHtR** |
| --- | --- | --- | --- | --- |
| **low BMI and low WHtR** | - |  |  |  |
| **high BMI and low WHtR** | < 0.001 | - |  |  |
| **low BMI and high WHtR** | <0.001 | <0.001 | - |  |
| **high BMI and high WHtR** | < 0.001 | <0.001 | 0.399 | - |

Benjamini-Hochberg method for adjusting significance level; The six p values (i.e., 0.0083, 0.01, 0.0125, 0.0167, 0.025, and 0.05) are judged “significant” by Benjamini-Hochberg procedure.

BMI, body mass index; WHtR, waist-to-height ratio.

**Supplementary** Fig. S1 Kaplan-Meier curves of the exposed (high-value) and non-exposed (low-value) groups for incident diabetes

The Kaplan-Meier curves of the exposed and non-exposed groups did not cross during the follow-up time, i.e., one curve was always above the other curve, which means the violation of proportionality was not extreme, and a single HR for the exposures can still be a reasonable summary of the data

1. BMI


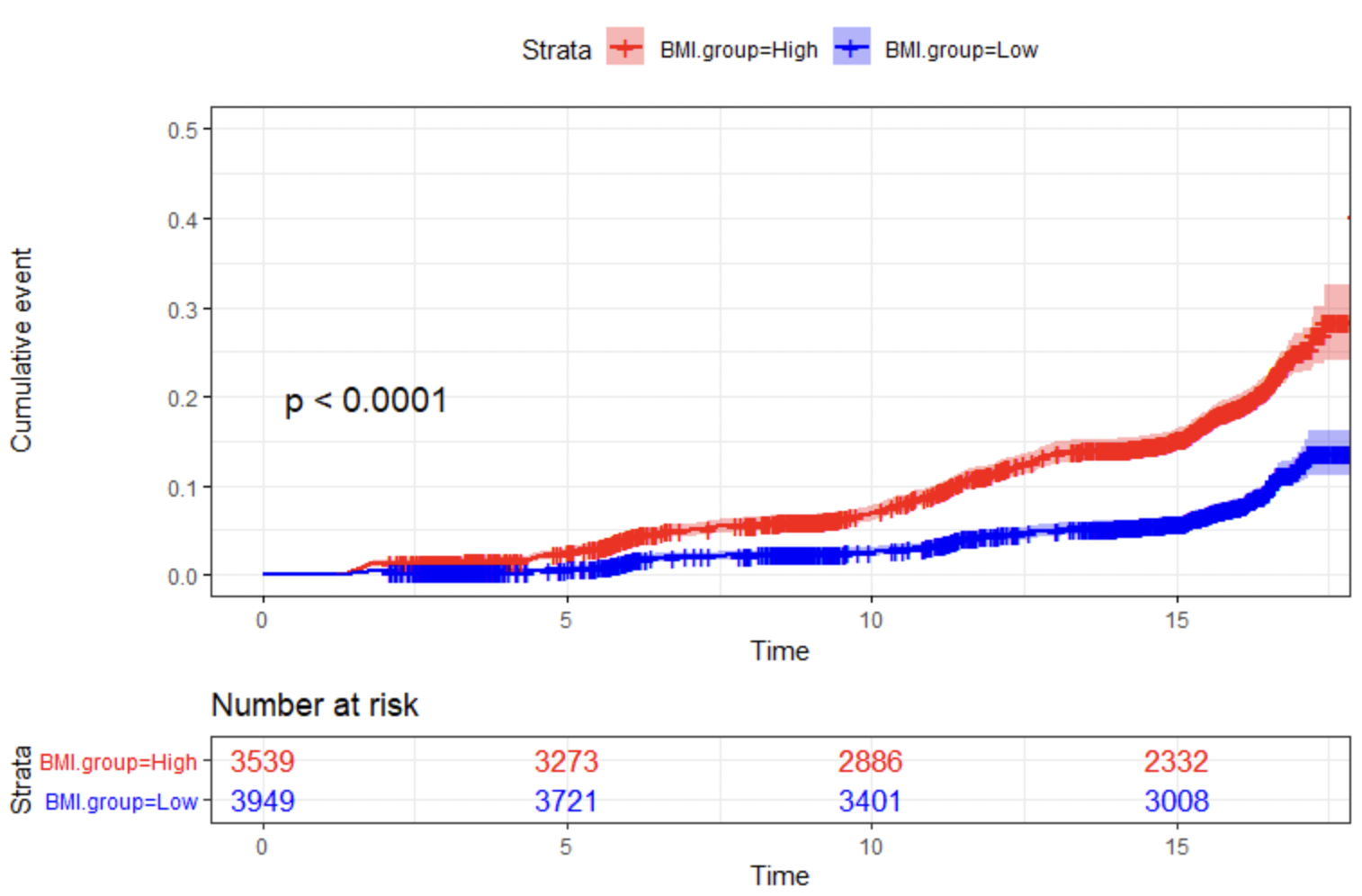


Time: time after Phase 3 (year 0) in years;

BMI, body mass index

1. WC


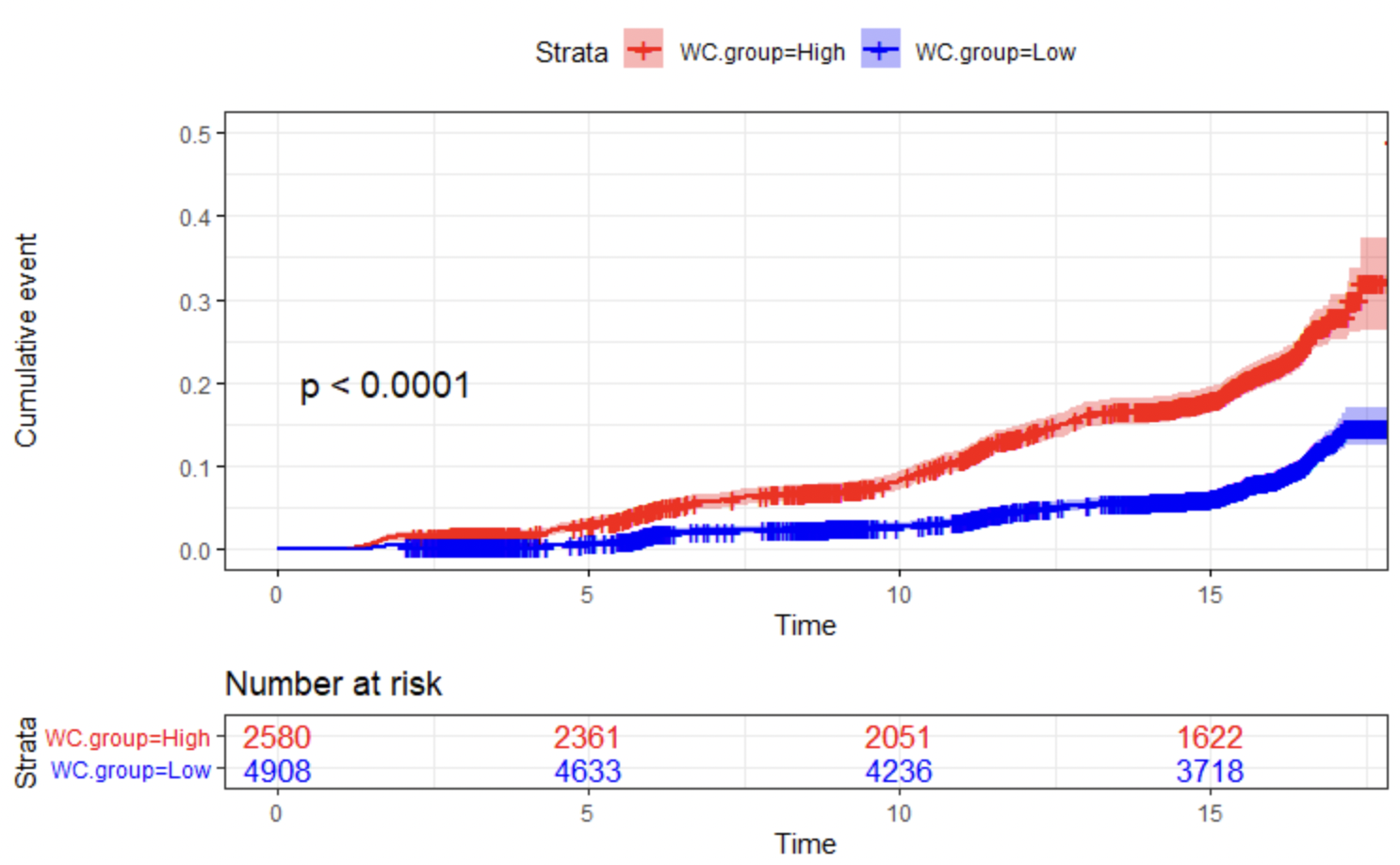


Time: time after Phase 3 (year 0) in years;

WC, waist circumference

1. WHtR


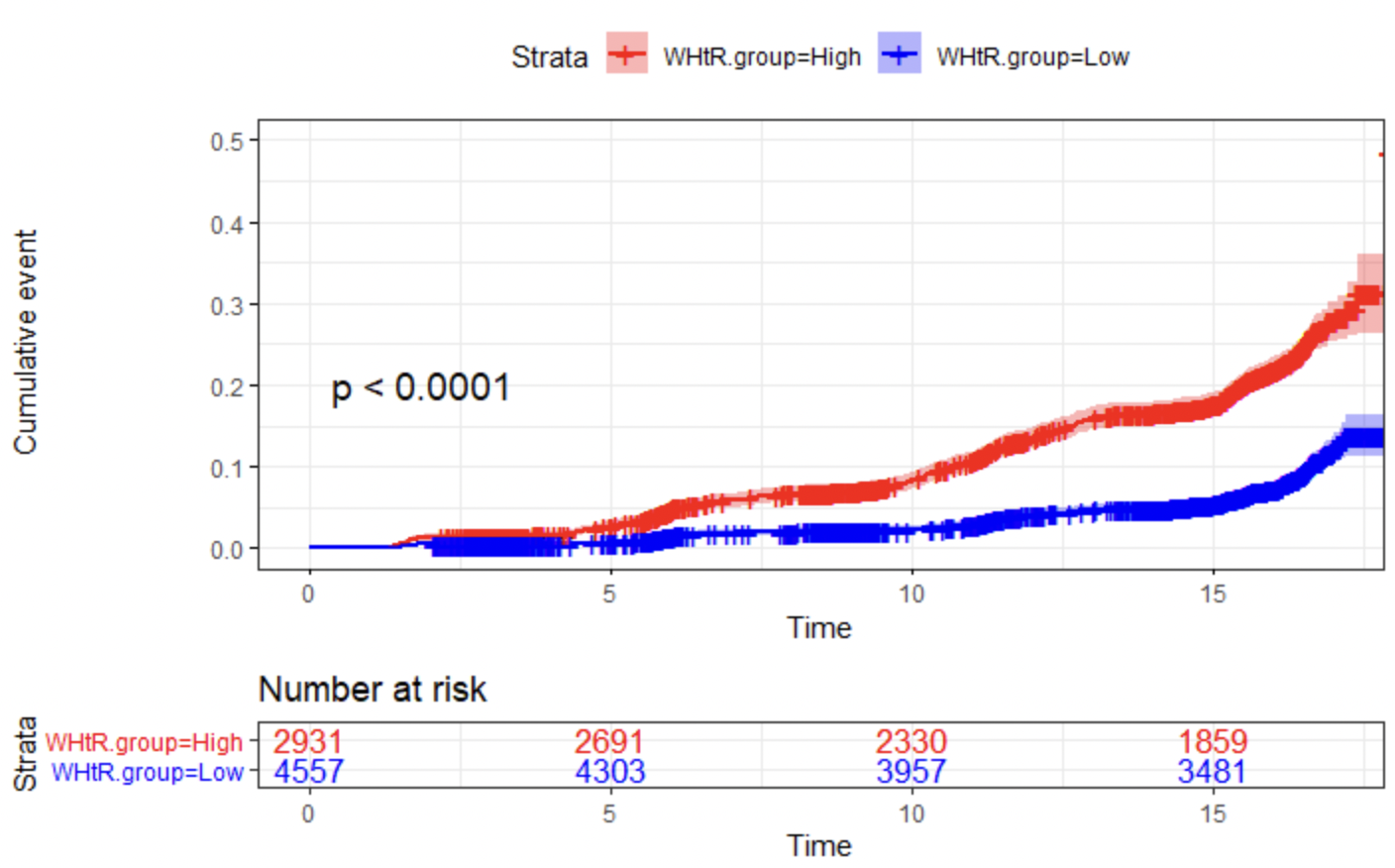


Time: time after Phase 3 (year 0) in years;

WHtR, waist-to-height ratio

1. WHT.5R


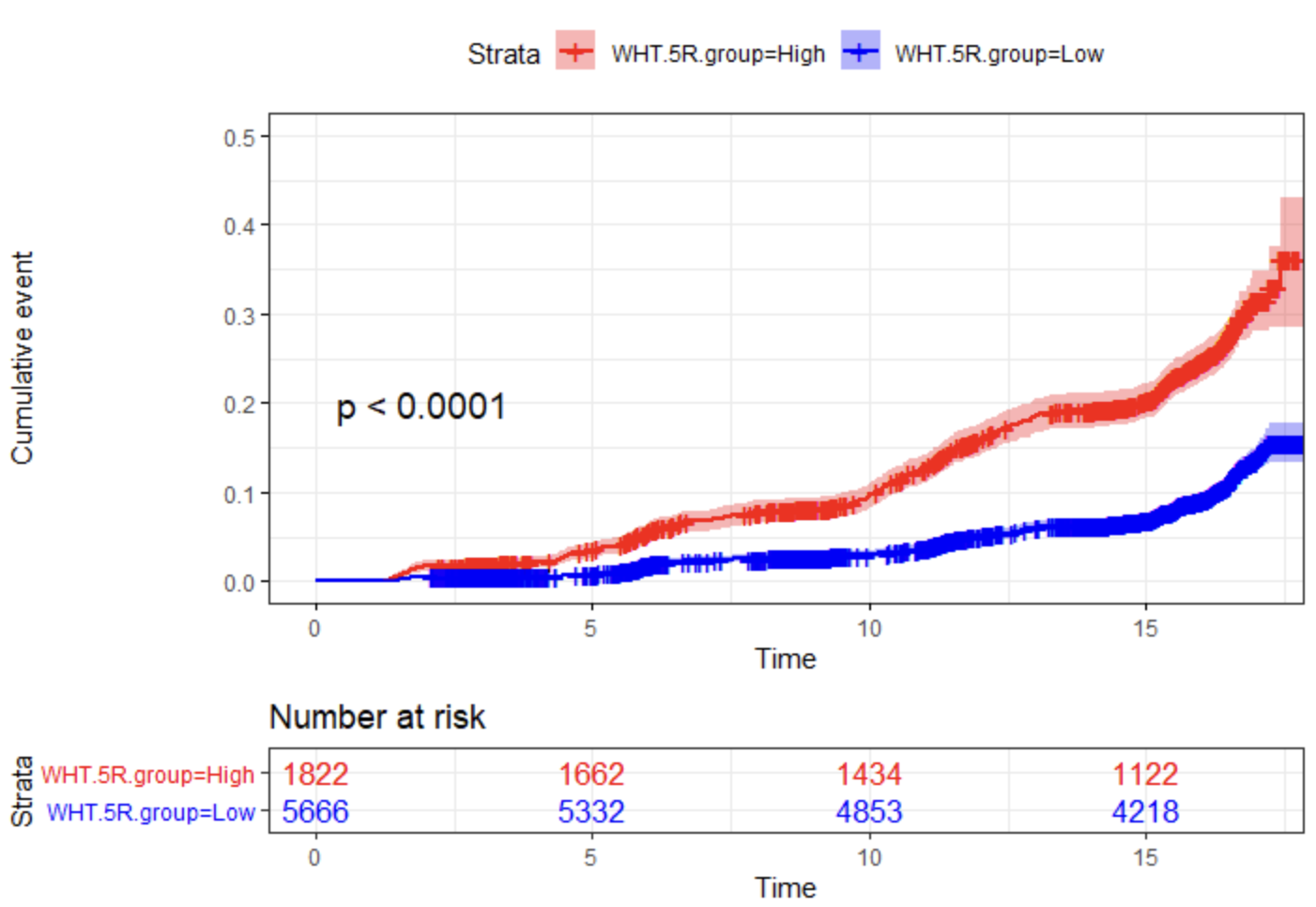


Time: time after Phase 3 (year 0) in years;

WHT.5R, waist-by-height^0.5^

1. ABSI


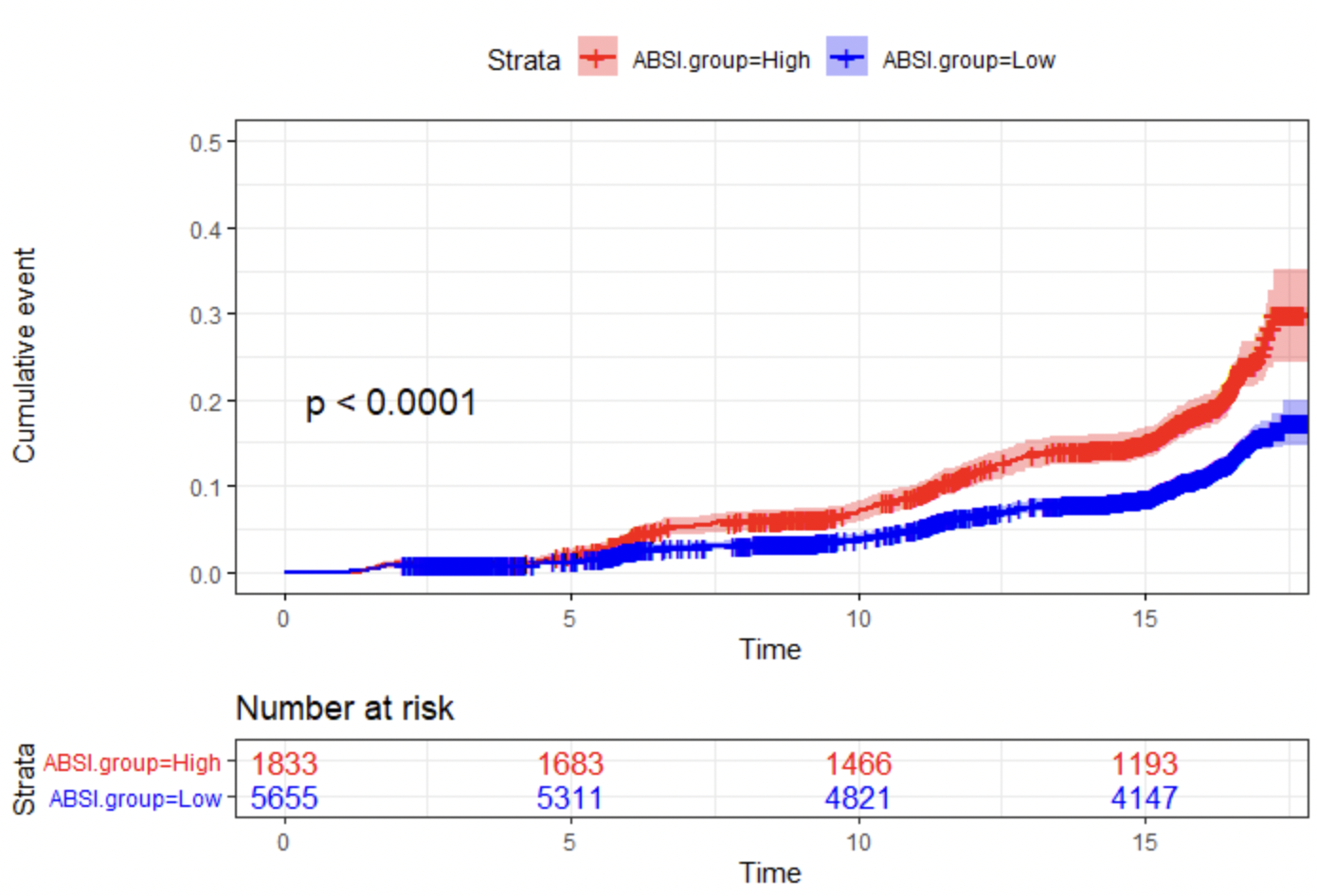


Time: time after Phase 3 (year 0) in years**;**

ABSI, a body shape index

**Supplementary** Fig. S2 Graphs of the scaled Schoenfeld residuals against the transformed time for the five exposure variables

In the graphs, the coefficients of the exposure variables did not change significantly during the follow-up period, i.e., Beta(t) for exposure was nearly constant over time (there is also no significant tendency to rise and then fall, or to fall and then rise), which means the violation of proportionality was not extreme, and a single HR for the exposures can still be a reasonable summary of the data.

1. **BMI**

**
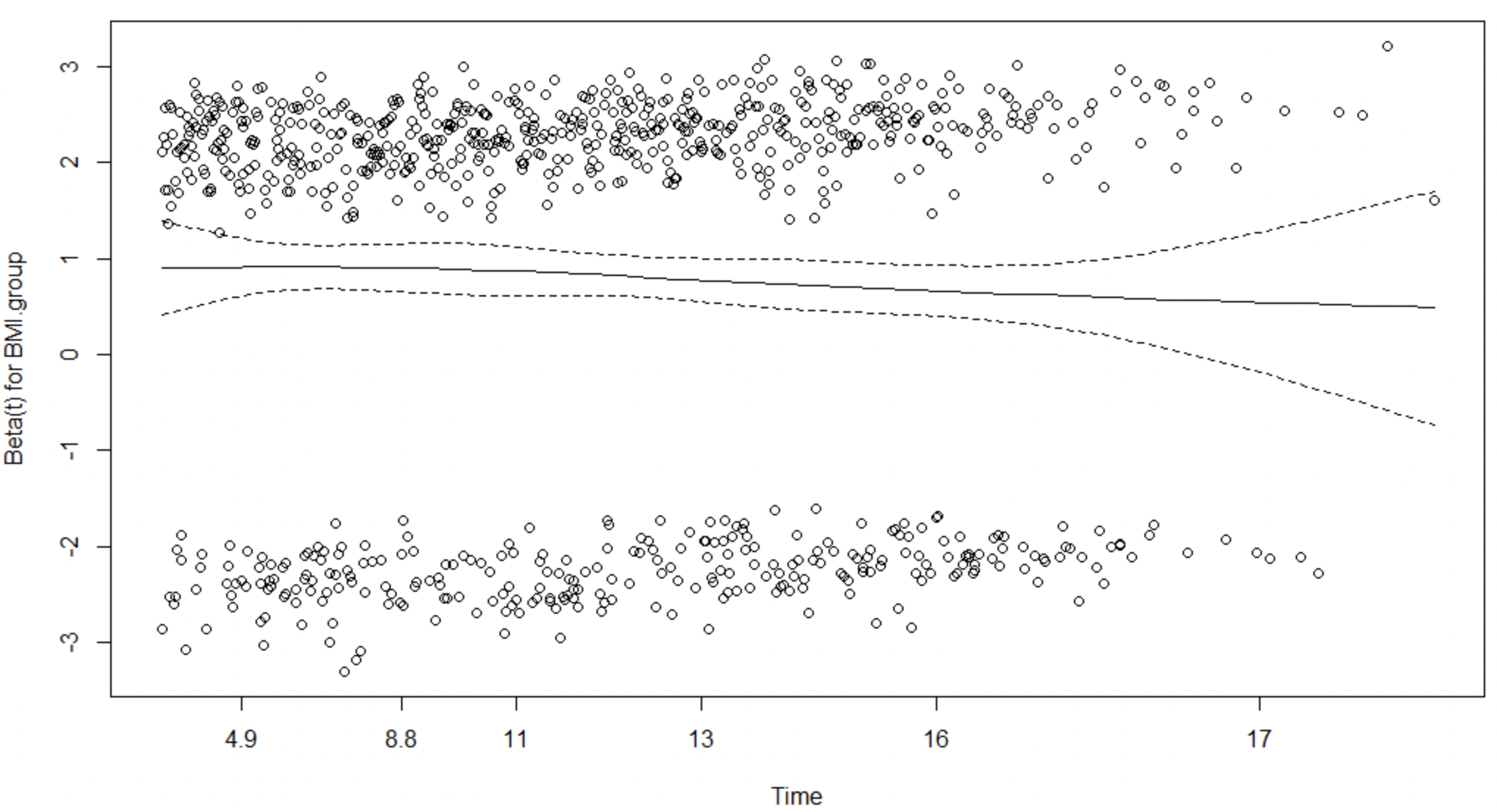
**

1. **WC**

**
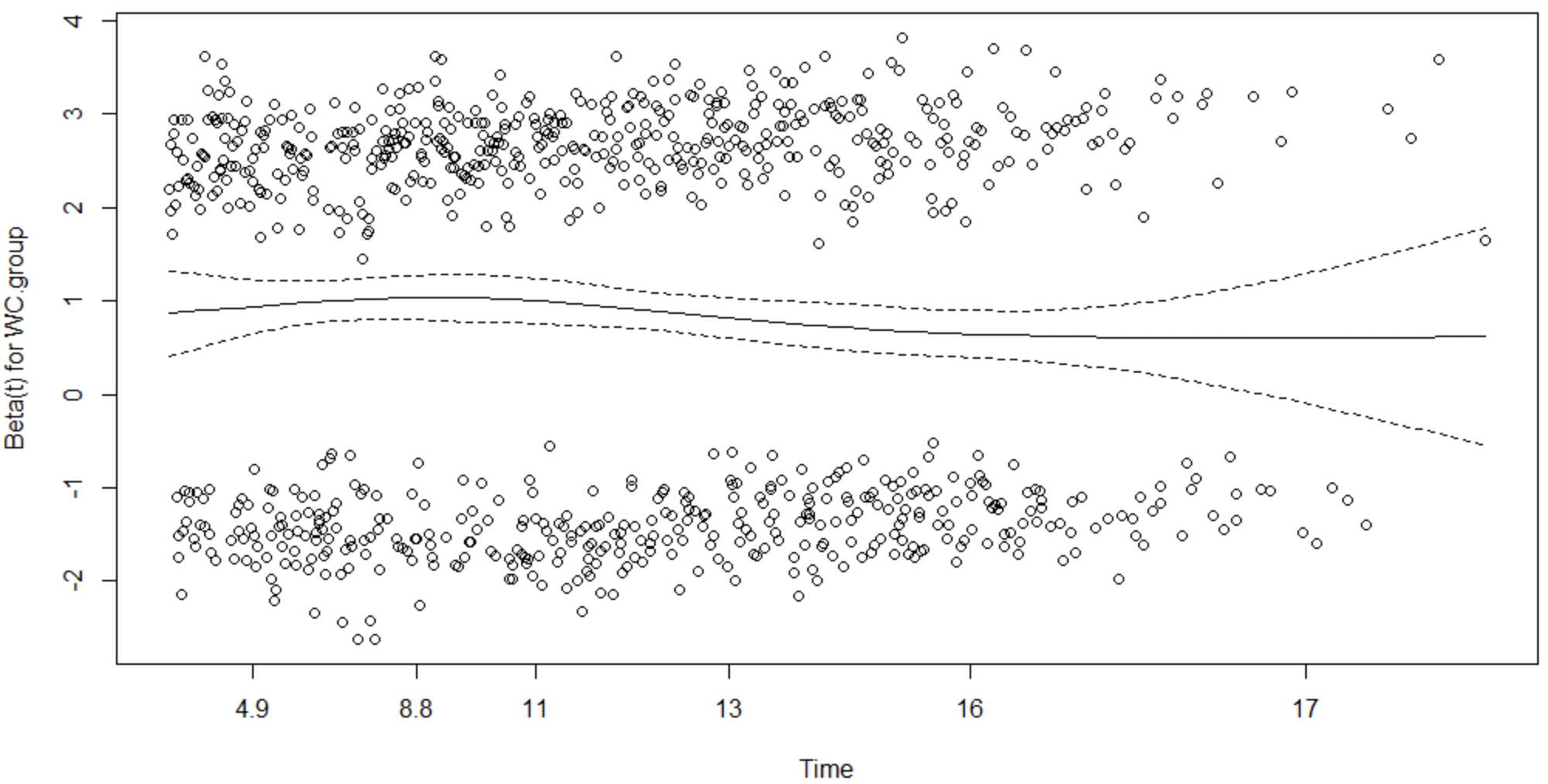
**

1. **WHtR**

**
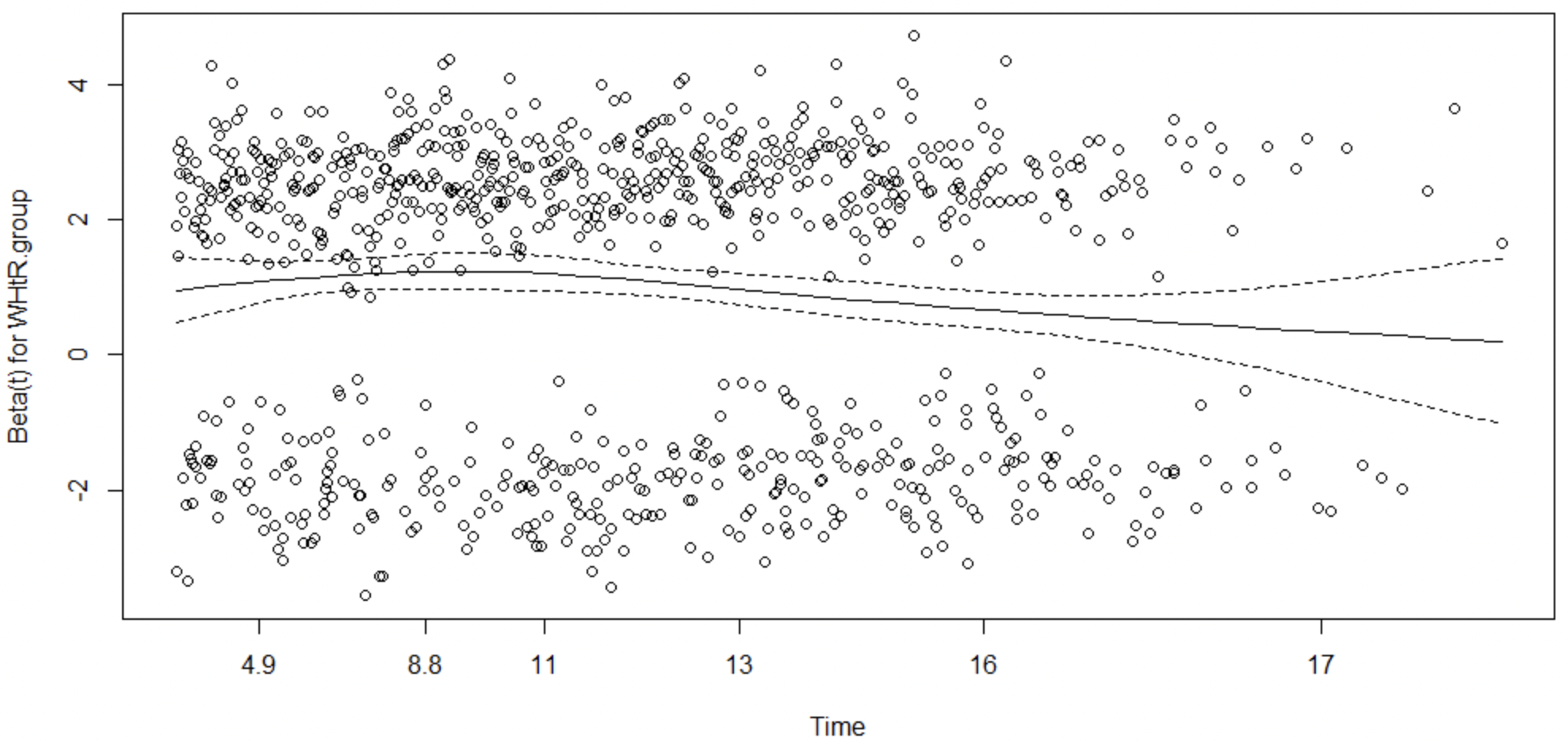
**

1. **WHT.5R**

**
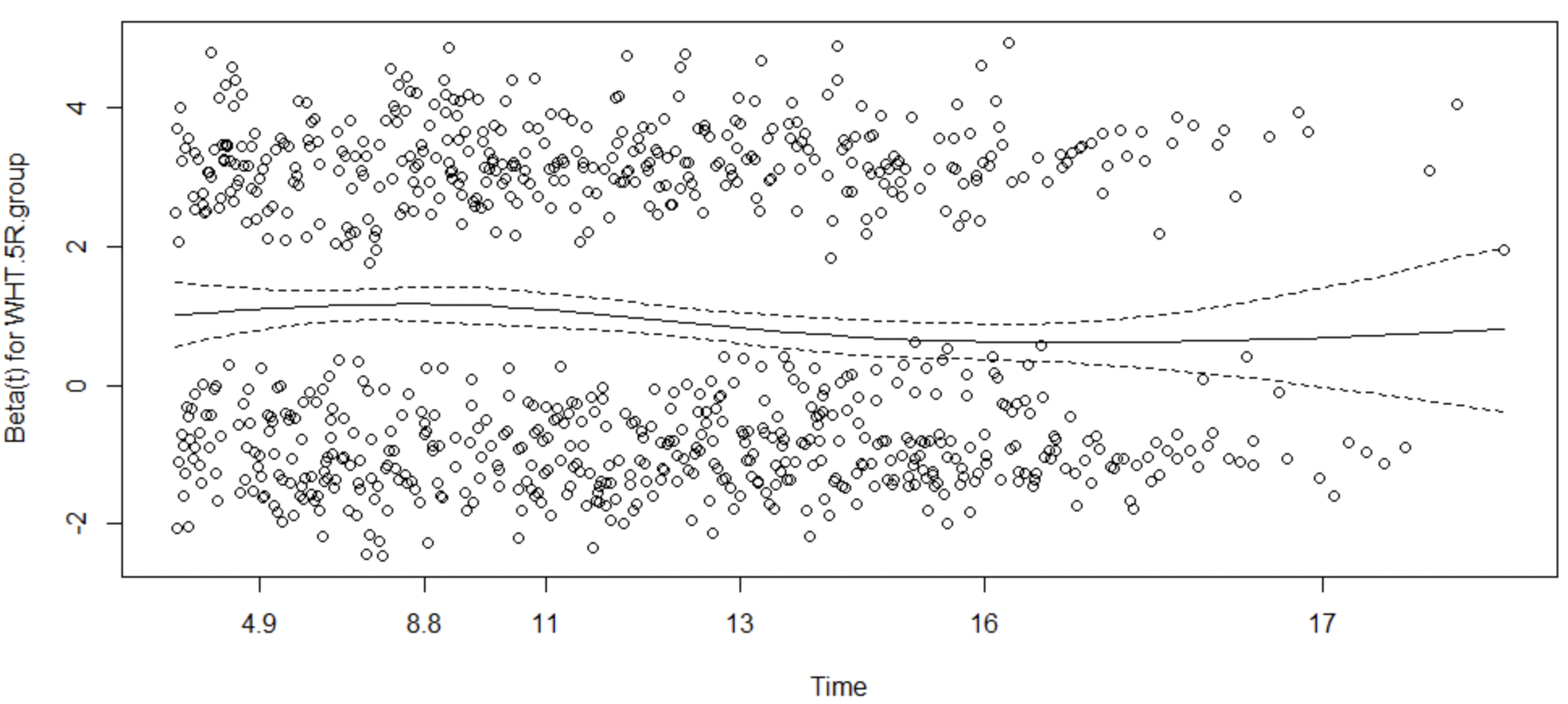
**

1. **ABSI**

**
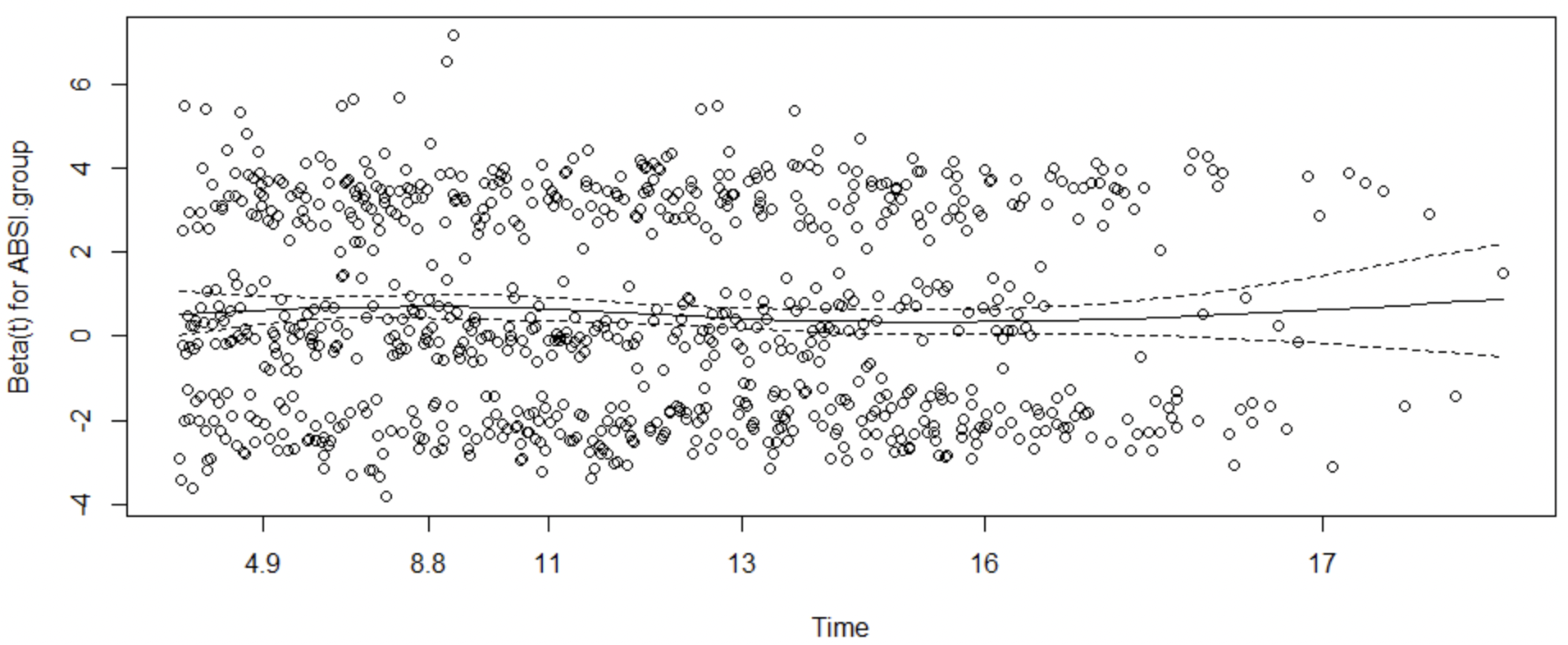
**

Time: time after Phase 3 (year 0);

BMI, body mass index; WC, waist circumference; WHtR, waist-to-height ratio; WHT.5R, waist-by-height^0.5^; ABSI, a body shape index

**Supplementary** Fig**. S3** Kaplan-Meier curves for incident diabetes in relation to different levels of BMI and WHtR combinations

1. Among Sample II (N=7488)


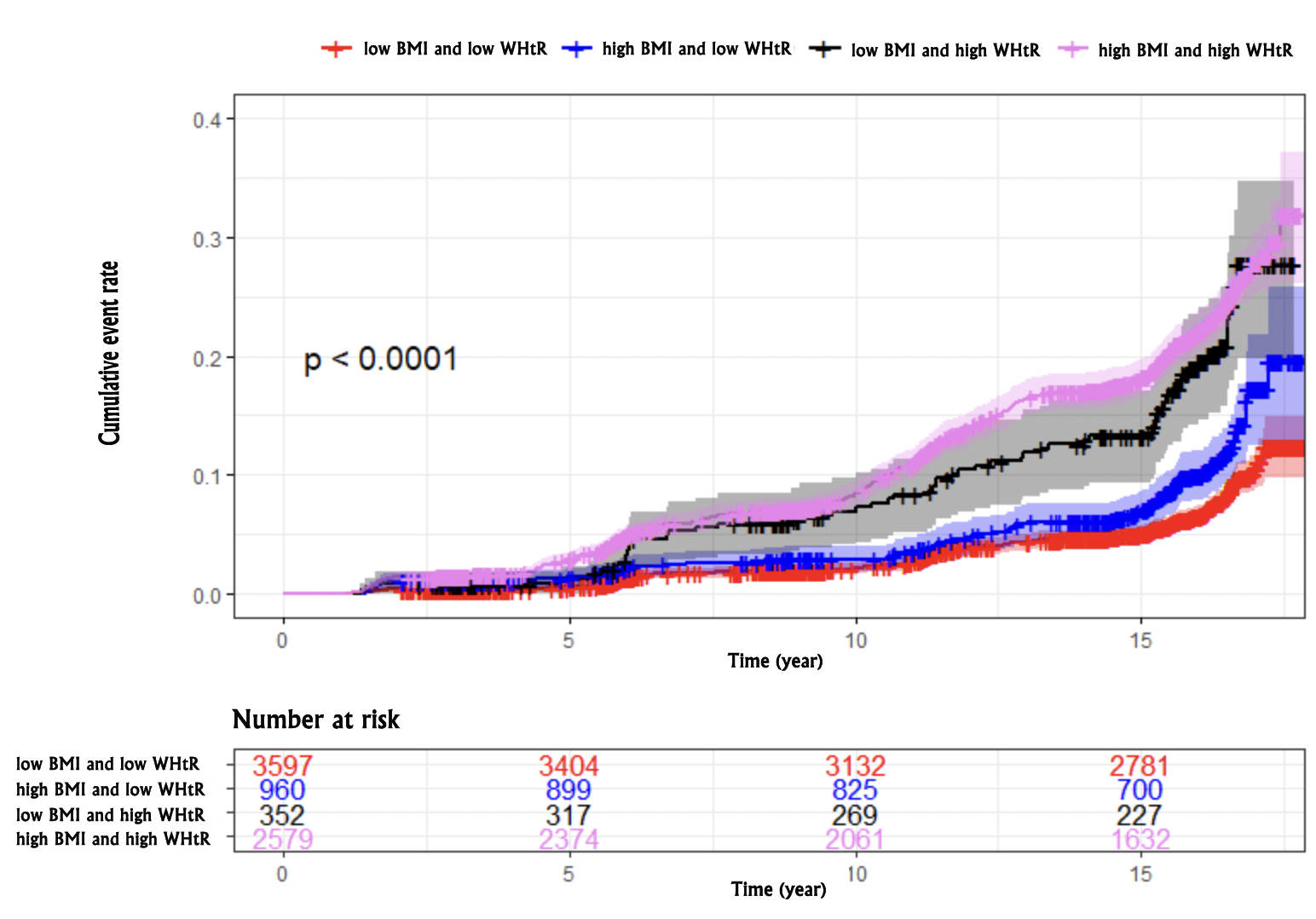


1. Among the persons with complete data of Sample II (N=6813)


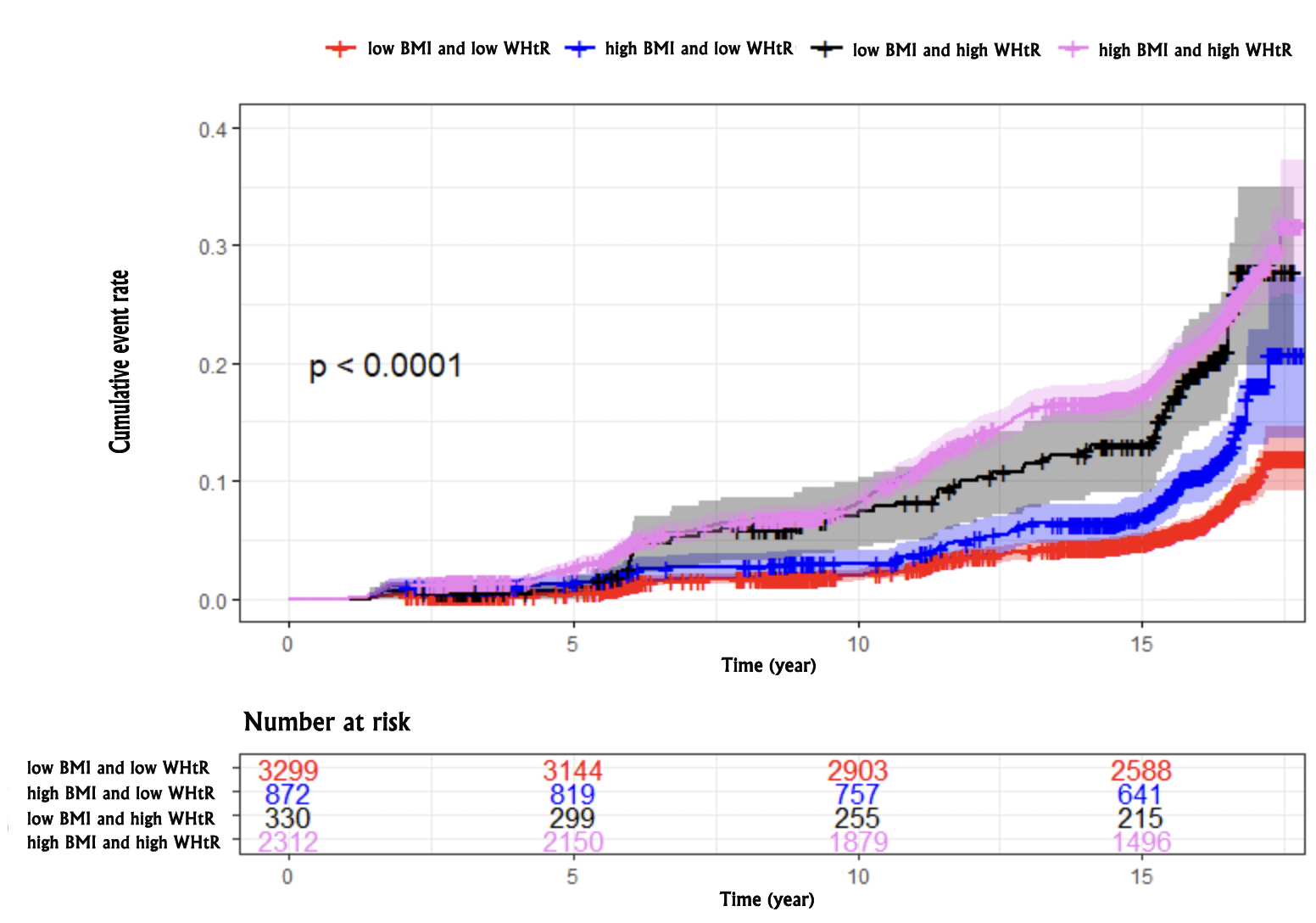


Time: time after Phase 3 (year 0)

BMI, body mass index; WHtR, waist-to-height ratio.
